# Supplementary material for: Vibrational Energy Dissipation in Noncontact Single-Molecule Junctions Governed by Local Geometry and Electronic Structure
Source: JACS Au. 2025 Sep 15;5(10):5027–38. doi: 10.1021/jacsau.5c00931 (PMC12569693; doi:10.1021/jacsau.5c00931)
Supplement: Supplementary file 1 [file au5c00931_si_001.pdf]

Supporting Information

Vibrational Energy Dissipation in Non-Contact Single-Molecule Junctions Governed by Local Geometry and Electronic Structure

Lukas Hörmann*,†,‡ and Reinhard J. Maurer*,†,‡

†*Department of Chemistry, University of Warwick, Gibbet Hill Rd, Coventry, CV4 7AL, UK*

‡*Department of Physics, University of Warwick, Gibbet Hill Rd, Coventry, CV4 7AL, UK*

E-mail: lukas.hoermann@warwick.ac.uk; r.maurer@warwick.ac.uk

Contents

S1 DFT calculations **3**

S1.1 Lattice constant convergence . . . 3

S1.2 K-grid convergence . . . 3

S1.3 Selecting the functional . . . 4

S1.4 Cu(111) adsorption environments . . . 5

S1.5 The Hessian and vibration frequencies of the CO molecule . . . 6

S1.6 Electron-phonon coupling convergence . . . 8

S1.7 Electronic friction tensors . . . 10

S1.7.1 Electron-phonon coupling of a CO molecule far away from the surface . . . 11

S2 Machine-learned interatomic potentials **12**

S2.1 Hyperparameter tuning . . . 12

S2.2 Testing the final potential . . . 14

S3 Lifetimes from different methods **17**

S3.1 Electron-phonon coupling lifetimes from quasi-harmonic approximation . . . 17

S3.2 Lifetimes from equilibrium correlation analysis . . . 17

S3.3 Lifetimes from kinetic energy decay . . . 22

S3.4 Lifetimes from additive relaxation rates . . . 25

S3.5 Comparison of lifetimes from different methods . . . 26

S3.6 Contributions to the relaxation rates . . . 27

S4 Comparison to other coinage metals **28**

S4.1 Vibrational properties and phonon-phonon coupling . . . 28

S4.2 Electron-phonon coupling . . . 29

S5 Analysis of the velocity cross-correlation function **30**

S6 Coupling strength between vibrational modes **31**

S6.1 Vibrational modes and amplitudes . . . 32

S6.2 Cross-correlation function . . . 35

S6.3 Coupling strength between vibration modes . . . 36

References **38**

S1 DFT calculations

For all DFT calculations, we use the electronic structure code FHI-aims[1] and numerical atom-centred basis functions. Training data for machine-learned interatomic potentials (MLIP) was created using version 240403, while electron-phonon coupling (EPC) calculations were done with version 200428.840. Both versions were regression tested to ensure that results were consistent. We first converge the lattice constant. Next, we benchmark functionals with respect to the predicted adsorption site. Then, we compare the computed vibration frequencies to experiment. Finally, we converge the lifetimes resulting from EPC with respect to the numerical settings.

S1.1 Lattice constant convergence

We converge the lattice constant using a primitive bulk unit cell, with periodic boundary conditions and a k-grid with 24 k-points in each direction. Sweeping the volume around the experimental lattice constant and fitting this data with a Birch–Murnaghan function allows us to determine the lattice constant. We find 2.543 Å and 2.545 Å for PBE+MBD-NL and HSE06+MBD-NL respectively (see Figure S1). For all calculations, we use tight default basis functions.

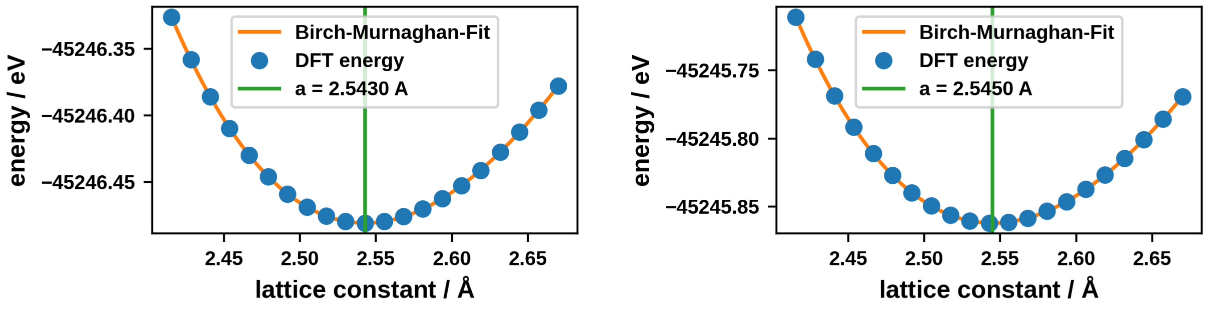

Figure S1: Lattice constant convergence; (left) PBE+MBD-NL; (right) HSE06+MBD-NL

S1.2 K-grid convergence

Using the lattice constant of 2.543 Å and 2.545 Å for PBE+MBD-NL and HSE06+MBD-NL, respectively, we converge the k-grid. We converge the total energy of a bulk Cu crystal since the data with these settings (at HSE06+MBD-NL level) is intended to train MLIPs. Figure S2 shows the k-grid convergence of the total energy. We find that k-grids with 16 and 24 k-points along each reciprocal lattice vector of the primitive bulk unit cell converge the total energy to within 1 meV for PBE+MBD-NL and HSE06+MBD-NL, respectively. Therefore, we use k-grids equivalent to 24 k-points along each reciprocal lattice vector for all HSE06+MBD-NL with periodic boundary conditions.

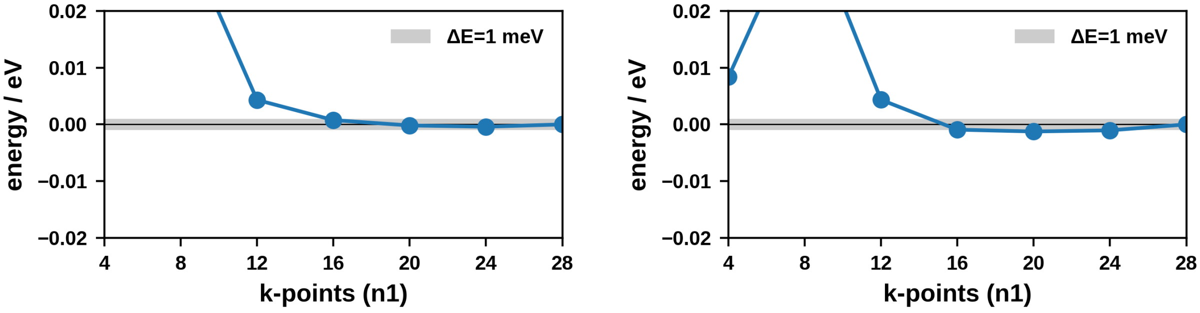

Figure S2: K-grid convergence of the total energy of the primitive unit cell of bulk copper; (left) PBE+MBD-NL; (right) HSE06+MBD-NL

S1.3 Selecting the functional

It is well known that the electronic structure of CO on various metal surfaces is difficult to describe using semi-local DFT.[2–4] In particular, common GGA functionals fail to predict the correct adsorption site, which is attributed to the many-electron self-interaction error. For the present work, it is necessary to use a computation method that yields a qualitatively correct description of the adsorption sites and vibration modes of CO on Cu(111). We test a number of different GGA, meta-GGA, and hybrid functionals in combination with common vdW-correction schemes and compare the computation results to adsorption energies determined using thermal desorption spectroscopy. Table S1 shows that the meta-GGA functional SCAN and the HSE06 short-range separated hybrid functional yield qualitatively correct adsorption energies. We use periodic boundary conditions and the repeated slab approach. The k-grid is equivalent to a (24, 24, 1) k-grid for a primitive surface unit cell.

Table S1: Adsorption energies for CO on Cu(111); *geometry of HSE06+MBD-NL.

**functional**	**adsorption energy / eV**	
	**top**	**fcc hollow**
experiment	-0.46,[5] -0.49[6]	-
rPBE+vdW^{surf}	-0.723	-0.781
PBE+MBD-NL	-0.967	-1.122
SCAN	-1.007	-0.930
HSE06+MBD-NL	-0.817	-0.761
HSE06*	-0.600	-0.536

The DFT adsorption energy is an approximation for energies measured with thermal desorption spectroscopy since the DFT energy does not account for zero-point vibrations and finite temperature effects. Therefore, the DFT energies should be seen as upper bounds for the magnitude of the adsorption energy. We included zero-point vibrations for a better comparison to the experiment. Using PBE+MBD-NL, the zero point energies are 0.379 eV and 0.360 eV for the top and fcc-hollow sites, respectively. For HSE06+MBD-NL, the zero-point energies are 0.395 eV and 0.357 eV for the top and fcc-hollow sites, respectively. For CO the zero-point energy is 0.264 eV and 0.277 eV, for PBE+MBD-NL and HSE06+MBD-NL, respectively. Accounting for zero-point energies reduces the total binding energy and brings it closer to the experimental values as shown in Table

S2. While the energy difference between the top and hollow sites is decreased in both PBE+MBD-NL and HSE06+MBD-NL, the top site remains more energetically favorable in case of HSE06+MBD-NL.

Table S2: Adsorption enthalpies of CO on Cu(111) in eV.

**functional**	**adsorption enthalpy / eV**	
	**top**	**fcc hollow**
experiment	-0.46,[5] -0.49[6]	-
PBE+MBD-NL	-0.852	-1.026
HSE06+MBD-NL	-0.699	-0.681

Additionally, we check the site preference for Cu-adatoms (see Table S3) and CO adsorbed on such adatoms (see Table S4) using HSE06+MBD-NL. We find that the fcc-hollow site is preferred over the bridge site, as reported in the literature.[7]

Table S3: Adsorption energy of Cu adatom.

	**total energy / eV**	**relative energy / eV**
fcc hollow	-4388827.986	0
bridge	-4388827.944	0.042

Table S4: Adsorption energy of CO on Cu adatom.

	**total energy / eV**	**relative energy / eV**
fcc hollow	-4391913.640	0
bridge	-4391913.605	0.035

We find that HSE06+MBD-NL yields qualitatively correct adsorption sites and therefore choose to proceed with this functional.

S1.4 Cu(111) adsorption environments

We consider four surface shapes onto which a CO molecule is placed: A sharp tip, a blunt tip, a (111)-slab with an adatom, and a (111)-slab. All geometries consist of a slab of four Cu layers that act as the base. On this slab, the different tip geometries are placed. In the case of the slab and the slab-adatom systems, this base slab is simply continued. The number of layers—counting also adatoms as a layer—of these geometries is given in Table S5.

Table S5: Number of substrate layers for each system.

**system**	**unmovable layers**	**movable layers**
sharp tip	4	10
blunt tip	4	0
slab adatom	4	11
slab	4	10

The lattice vectors of the unit cell for the sharp tip and the blunt tip are shown in Listing 1, while those of the slab and slab-adatom systems are shown in Listing 2.

```
lattice_vector 30.5159568199999995 0.0000000000000000 0.0000000000000000
lattice_vector -15.2579784099999998 26.4275938199999985 0.0000000000000000
lattice_vector -0.0000000000000000 -0.0000000000000000 86.2290435999999971
```

Listing 1: Lattice vectors of the sharp tip and blunt tip goemetries.

```
lattice_vector 15.2579784099999998 0.0000000000000000 0.0000000000000000
lattice_vector -7.6289892000000004 13.2137969099999992 0.0000000000000000
lattice_vector -0.0000000000000000 -0.0000000000000000 106.9925222699999949
```

Listing 2: Lattice vectors of the slab and slab-adatom goemetries.

The four adsorption environments contain 795, 793, 505, and 504 Cu atoms for the sharp tip, the blunt tip, the slab-adatom, and the slab, respectively. Figure S3 shows the different tip shapes.

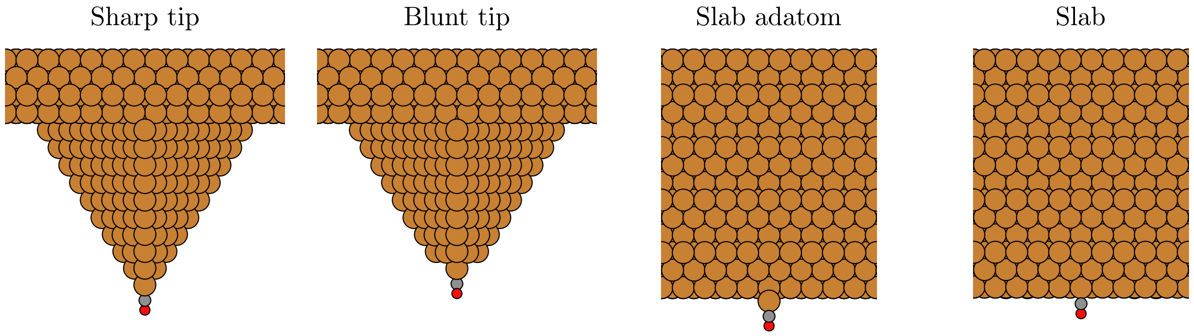

Figure S3: Different tip shapes considered in this study.

S1.5 The Hessian and vibration frequencies of the CO molecule

CO on different Cu(111) geometries has four vibration modes, which are the frustrated translation (FT), the frustrated rotation (FR), the metal-CO stretch (MS), and the internal stretch (IS). We calculate the vibration frequencies with DFT. We do this to determine the second-order force constants, which are the elements of the Hessian.

$$H_{\mathrm{ij}} = \frac{\partial^2 V}{\partial x_{\mathrm{i}} \partial x_{\mathrm{j}}} \tag{1}$$

Figure S4 shows a row in the Hessian, i.e. H_{ij} for a fixed i. The index i was chosen to be the x-axis degree of freedom of the C atom. The degrees of freedom are in no particular order. Each panel shows a different tip shape. The bars indicate the absolute value of the element H_{ij}. Large elements indicate strong coupling between the respective degrees of freedom.

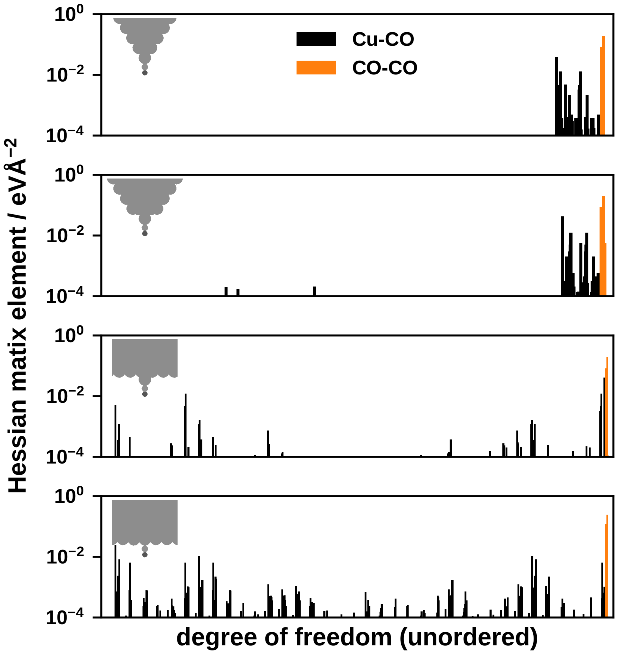

Figure S4: Off-diagonal elements of the Hessian.

Calculated vibrational frequencies are shown in Tables S7 and S8 for PBE+MBD-NL as well as HSE06+MBD-NL, respectively. Table S6 shows vibration frequencies determined by hydrogen scattering[8] and infrared spectroscopy.[9] The comparison of the three tables shows that PBE+MBD-NL and HSE06+MBD-NL yield vibrational frequencies for these modes that agree well with experiment.

Table S6: Vibration frequencies of a CO molecule on a surface from experiment.

	**vibrational energy / meV**			
	**FT**	**FR**	**MS**	**IS**
slab[8,9]	4.07	36.47	41.41	257.63

Table S7: Vibration frequencies of CO molecule on different geometries at PBE+MBD-NL level.

	**vibrational energy / meV**			
	**FT**	**FR**	**MS**	**IS**
sharp tip	3.25	31.69	49.51	254.80
blunt tip	3.56	34.23	51.13	254.68
slab top	5.29	35.46	47.87	250.19
slab fcc hollow	18.14	30.55	37.95	224.62

Table S8: Vibration frequencies of a CO molecule on different geometries at HSE06+MBD-NL level.

	vibrational energy / meV			
	**FT**	**FR**	**MS**	**IS**
sharp tip	2.9	29.07	45.44	271.97
blunt tip	2.94	31.21	45.63	272.16
slab adatom	3.27	32.57	45.32	274.12
slab top	5.49	36.5	44.68	266.21
slab fcc hollow	14.32	24.86	34.37	243.88

S1.6 Electron-phonon coupling convergence

To calculate EPC, we use a blunt Cu-tip placed on a Cu-slab made up of four layers. We use periodic boundary conditions and the repeated slab approach, with a dipole correction. The unit cell contains 144 slab surface atoms and has the following lattice vectors:

```
lattice_vector      30.51595682      0.00000000      0.00000000
lattice_vector     -15.25797841     26.42759382      0.00000000
lattice_vector       0.00000000      0.0000000      86.22904360
```

We converge the number of atom layers and the number of k-points. For Cu-atoms, we use light default basis functions, while we use tight defaults for all other species. Figure S5 shows the convergence of the number of layers in the tip with respect to the lifetime of the four non-degenerate vibration modes of the CO molecule adsorbed on the sharp Cu-tip. The number of layers in the slab is always four, and all calculations use the same unit cell. The convergence shows us that four slab layers + 9 layers in the Cu-tip are sufficient to converge the lifetime of the vibration modes.

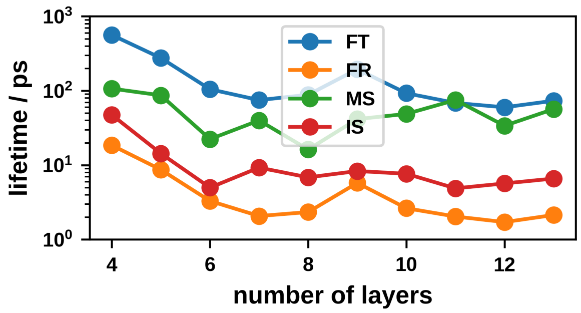

Figure S5: Layer convergence of the lifetime of the different CO vibration modes as a result of EPC for CO on a blunt tip

Figure S6 shows the convergence of the k-grid with respect to the lifetime of the non-degenerate vibration modes of the CO molecule on a surface slab. We use a (6, 6) unit cell to ensure that the CO molecule does not interact with its image. We test two different friction broadening vales of 0.1 eV and 0.3 eV (default setting). We find that a k-grid of (24, 24, 1) for the primitive substrate unit cell converges the lifetime. In the case of the (6, 6) unit cell, this k-grid would correspond to (4, 4, 1) k-points. Moreover, the default broadening of 0.3 eV shows better k-grid convergence.

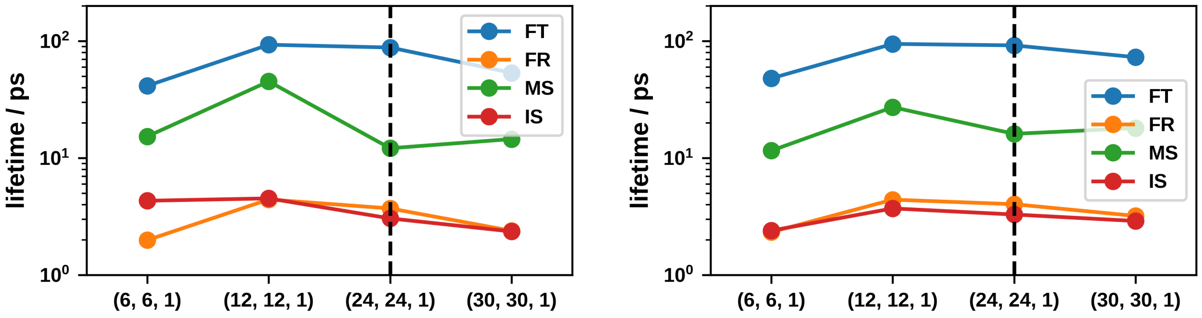

Figure S6: K-point convergence of the lifetime of the different CO vibration modes as a result of EPC for CO on a Cu(111) slab; left) friction broadening 0.1 eV; right) friction broadening 0.3 eV.

Figure S6 also shows better k-point convergence for a broadening of 0.3 eV. To gauge the effect of the friction broadening, we sweep the broadening in Figure S7. The broadening should be chosen as small as possible. We find that broadening values larger than the default of 0.3 eV do not significantly affect the lifetimes.

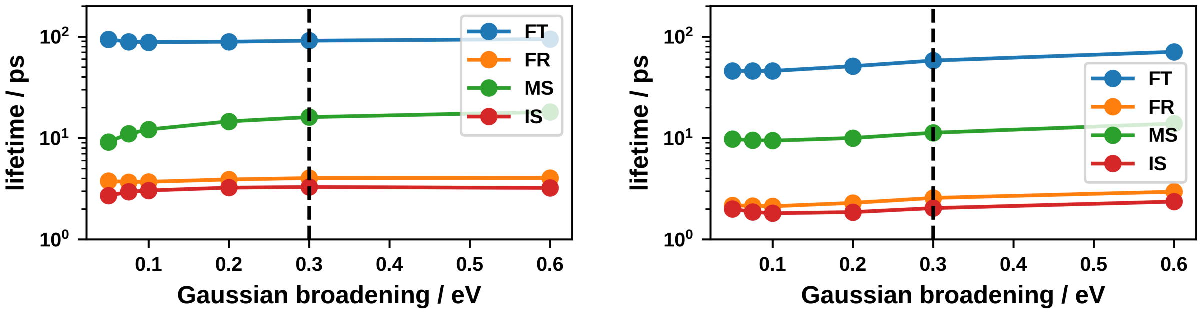

Figure S7: Dependence of the lifetime of the different CO vibration modes as a result of EPC for CO on a slab on the friction broadening; the k-grid is (24, 24, 1) for the primitive substrate unit cell; left) perturbing frequency of zero; right) perturbing frequency of the respective IS mode.

Below is a control file for the converged settings to calculate electron friction for the surface slab:

```
# General Settings:
xc                              pbe
spin                            none
charge                          0.0
relativistic                    atomic_zora scalar
occupation_type gaussian        0.01
k_grid                          4 4 1

# Convergence Criteria:
sc_accuracy_etot                1e-06
sc_accuracy_eev                 1e-03
sc_accuracy_rho                 1e-05
sc_iter_limit                   200

# Other Settings:
```

```
use_dipole_correction     # friction_tensor in 1/ps
# n_atom   1 i_cart   1 index   1
    0.014283   -0.000002   -0.000000
# n_atom   1 i_cart   2 index   2
   -0.000002    0.014285   -0.000001
# n_atom   1 i_cart   3 index   3
   -0.000000   -0.000001    0.014281    .true.
compensate_multipole_errors   .true.

# Friction keywords
calculate_friction            numerical_friction
friction_numeric_disp         0.001
friction_broadening_width     0.3
friction_temperature          5
friction_delta_type           gaussian
output friction_eigenvectors  .true.
friction_iter_limit           100
calculate_all_eigenstates
friction_delta_normalisation  .false.
friction_max_energy           2.4
```

S1.7 Electronic friction tensors

The entire electronic friction tensor is approximated by combining a friction tensor calculated for a CO molecule absorbed on the Cu surface with friction values calculated for the Cu bulk. For the Cu atoms, we use a diagonal electronic friction tensor, where electronic is homogeneous in all directions. The friction tensor for the adsorbed CO molecule is shown in Listing 3. The friction value for the Cu atoms is determined by calculating the electronic friction tensor for a Cu atom in the middle of a 5x5x5 Cu bulk unit cell. This electronic friction tensor is shown in Listing 4 and displays similar electronic friction coefficients in each degree of freedom.

```
  # friction_tensor in 1/ps
# n_atom 505 i_cart   1 index   1
    0.026131   -0.000003   -0.000003   -0.076470   -0.000009    0.000036
# n_atom 505 i_cart   2 index   2
   -0.000003    0.026078    0.000052    0.000009   -0.076360    0.000049
# n_atom 505 i_cart   3 index   3
   -0.000003    0.000052    0.055054    0.000074   -0.000071   -0.099630
# n_atom 506 i_cart   1 index   4
   -0.076470    0.000009    0.000074    0.229697    0.000005   -0.000213
# n_atom 506 i_cart   2 index   5
   -0.000009   -0.076360   -0.000071    0.000005    0.229491   -0.000015
# n_atom 506 i_cart   3 index   6
    0.000036    0.000049   -0.099630   -0.000213   -0.000015    0.313573
```

Listing 3: CO molecule adsobed on the Cu(111) slab.

```
  # # friction_tensor in 1/ps
# n_atom   1 i_cart   1 index   1
    0.014283   -0.000002   -0.000000
# n_atom   1 i_cart   2 index   2
```

```
   -0.000002    0.014285   -0.000001
# n_atom   1 i_cart   3 index   3
   -0.000000   -0.000001    0.014281
```

Listing 4: Cu atom in Cu bulk.

S1.7.1 Electron-phonon coupling of a CO molecule far away from the surface

We compare the electronic friction tensor of a CO molecule adsorbed on the Cu(111) slab to that of the CO molecule approximately 5 Å away from the slab. The adsorbed system, shown in Listing 3, shows electronic friction coefficients that are four orders of magnitude larger than the system where the molecule is at a distance, shown in Listing 5.

```
  # friction_tensor in 1/ps
# n_atom 505 i_cart   1 index   1
    0.000002    0.000000   -0.000000   -0.000000    0.000001   -0.000001
# n_atom 505 i_cart   2 index   2
    0.000000    0.000001   -0.000000    0.000000   -0.000000   -0.000000
# n_atom 505 i_cart   3 index   3
   -0.000000   -0.000000    0.000093   -0.000000    0.000000   -0.000105
# n_atom 506 i_cart   1 index   4
   -0.000000    0.000000   -0.000000    0.000002    0.000000   -0.000001
# n_atom 506 i_cart   2 index   5
    0.000001   -0.000000    0.000000    0.000000    0.000003   -0.000001
# n_atom 506 i_cart   3 index   6
   -0.000001   -0.000000   -0.000105   -0.000001   -0.000001    0.000124
```

Listing 5: CO molecule at a distance of 5 Å away from the Cu(111) slab.

S2 Machine-learned interatomic potentials

We use the MACE potential,[10] version 0.3.4, which is an equivariant neural-network potential. The training, test, and validation sets were chosen from a dataset containing 621 calculations performed at the HSE06+MBD-NL level of theory. This dataset includes structures sampled from molecular dynamics (MD) simulations using lower-accuracy MLIPs, configurations with randomly perturbed atomic positions, structures obtained during geometry optimisation, and vibration calculations. The structures in the training set range from 79 Cu atom clusters to a CO molecule adsorbed on a 796-atom Cu surface geometry. To find the best potential, we determine the optimal cutoff distance (r_{max}) and the message size (hidden_irreps). For each combination of cutoff distance and message size, we train five potentials, evaluate the training and validation errors, and determine the mean value of these errors. Figures S8 and S9 show the results for energies and forces, respectively. Because these MLIPs are intended to run MD simulations, we place greater emphasis on accurate forces when selecting the parameters.

S2.1 Hyperparameter tuning

We perform a hyperparameter search to find optimal model parameters that deliver minimal root mean square errors (RMSE) and mean absolute errors (MAE) on the test set and provide robust models in terms of cross-validation. We test different combinations of cutoff distance (r_{max}) and the message size (hidden_irreps). For each combination of cutoff distance and message size, we trained five potentials, evaluated the training and validation errors, and determined the mean value of these errors. For this, we use k-fold cross-validation, where we split our dataset into five equally sized segments. Four segments are used for training, while the final segment is split into a validation and a test set. We use the validation set for hyperparameter selection. Figures S8 and S9 show the averaged errors for energies and forces, respectively. Because these MLIPs are intended to run MD simulations, we place greater emphasis on accurate forces when selecting the parameters.

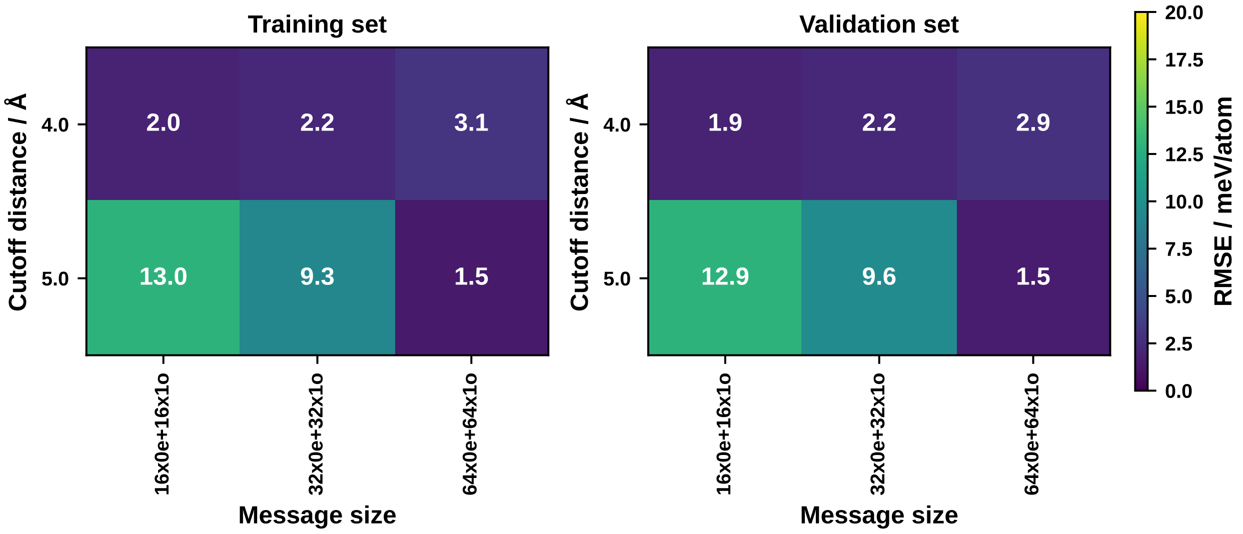

Figure S8: Average energy error for different MACE potentials.

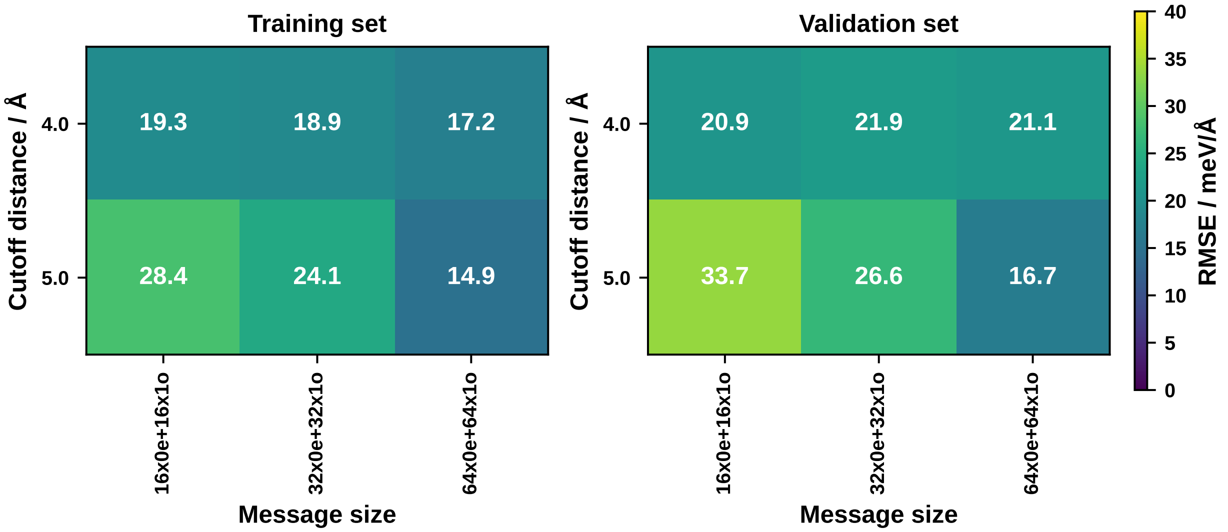

Figure S9: Average force error for different MACE potentials.

When analysing the force errors in Figure S10, we find that a cutoff radius 4.0 Å or 5.0 Å leads to the smallest training and validation set errors. Moreover, the errors decrease with increasing message size. To select the final production training set, we determine the potentials with the lowest training and validation errors. Figure S10 shows the potential with the smallest force error for each combination of parameters.

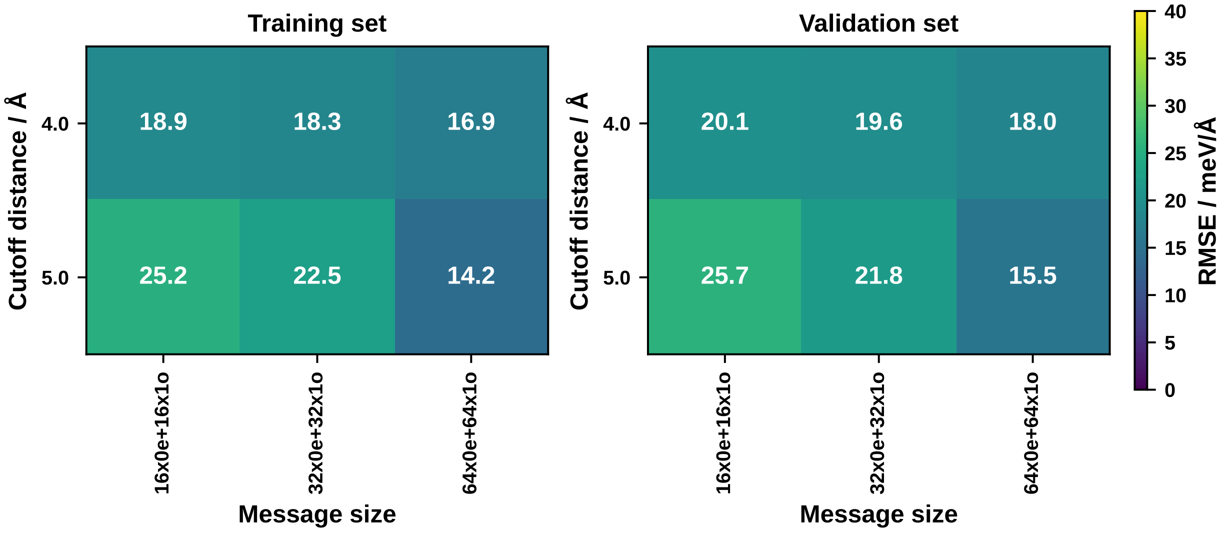

Figure S10: Smallest force errors achieved for each combination of cutoff distance and the message size.

We find that a cutoff radius of 5.0 Å combined with a message size of $64x0e + 64x1o$ yields the best MLIP. The following code section shows the final parameters for the MACE potential.

```
name = MACE_model
train_file = data/train.xyz
valid_file = data/valid.xyz
test_file = data/test.xyz
config_type_weights = {"Default":1.0}
E0s = {6:-0.102674349116667E+04, 8:-0.203970920939905E+04, 29:-0.452419011191033E+05}
model = MACE
hidden_irreps = 64x0e+64x1o
r_max = 5.0
batch_size = 10
max_num_epochs = 1500
swa = True
start_swa=1200
ema = True
ema_decay=0.99
amsgrad = True
restart_latest = True
device = cuda
save_cpu = True
forces_weight = 95
swa_forces_weight = 1
energy_weight = 5
swa_energy_weight = 10
correlation = 2
num_interactions = 2
num_radial_basis = 3
num_cutoff_basis = 4
default_dtype = float64
```

Using the five training, test, and validation sets described earlier, we quantified uncertainties on the test set based on the final model parameters (a cutoff radius of 5.0 Å and a message size of $64x0e + 64x1o$). Table S9 presents the average errors across the five sets for all atoms, along with the force errors specifically for the O atom in the CO molecule and the Cu atoms.

Table S9: Averaged errors for energies and forces for five different samples.

	Energy / meV/atom		Force / meV Å^{-1}	
Species	RMSE	MAE	RMSE	MAE
Cu	-	-	15.7 (2.0)	10.0 (0.9)
O	-	-	32.6 (9.3)	16.8 (3.8)
**All**	**1.6 (0.2)**	**1.2 (0.2)**	**15.9 (1.9)**	**10.0 (0.9)**

S2.2 Testing the final potential

As the final model, we chose the model that yielded the smallest force error in Figure S10. To test the accuracy of our final MACE potential, we determine learning curves for energies and forces, which are shown in Figure S11. Potentials were trained using training sets containing 150, 200, 250, 300, 350, 400, 450, and 504 calculations. The smaller training sets were randomly selected from the set of 504 data points. The training was done using a validation set containing 69 calculations. We evaluated all models on the test set containing 48 calculations. As expected, larger training sets lead to a clear improvement in prediction

accuracy across all data sets, and we are therefore confident in the validity of our final MACE potential. The errors of the final potential trained on 504 data points are shown in Table S10.

Table S10: Errors for energies and forces for the final potential trained on 504 data points.

	Energy / meV/atom		R^2	Force / meV Å^{-1}		R^2
Species	RMSE	MAE		RMSE	MAE	
Cu	-	-	-	15.6	9.6	0.995
O	-	-	-	27.7	16.2	0.998
**All**	**2.0**	**1.5**	**1.000**	**15.7**	**9.6**	**0.996**

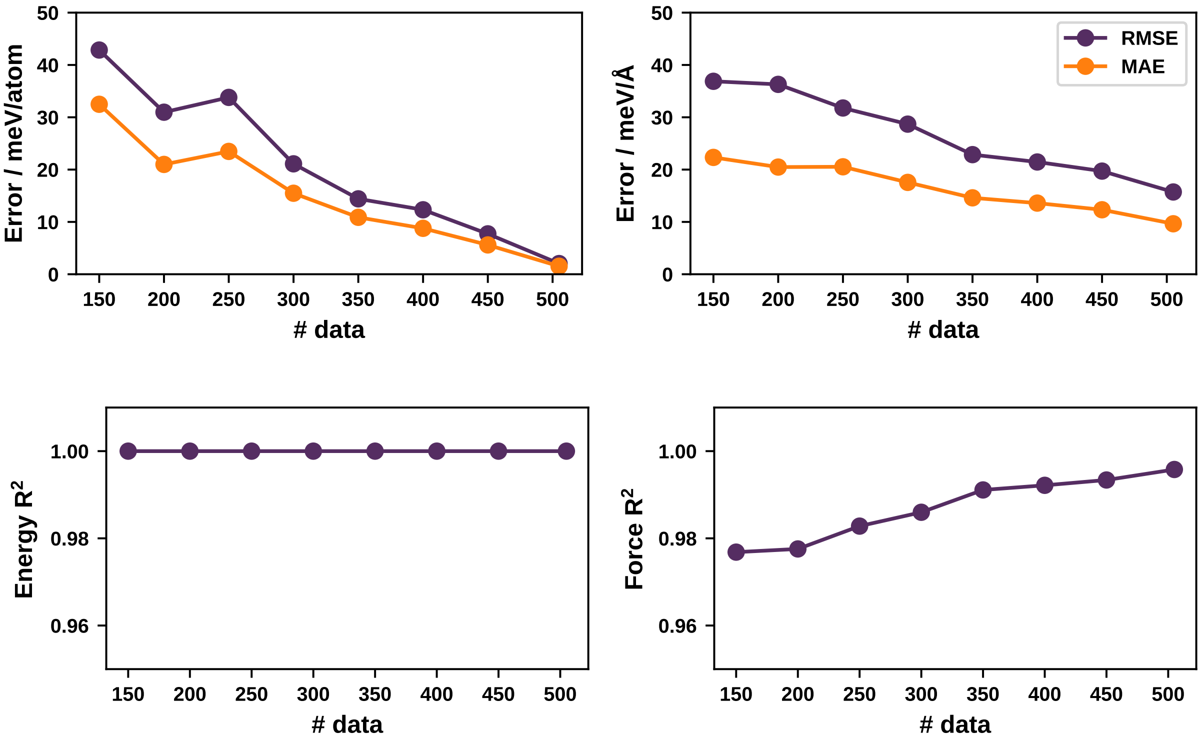

Figure S11: Learning curve for the final MACE potential

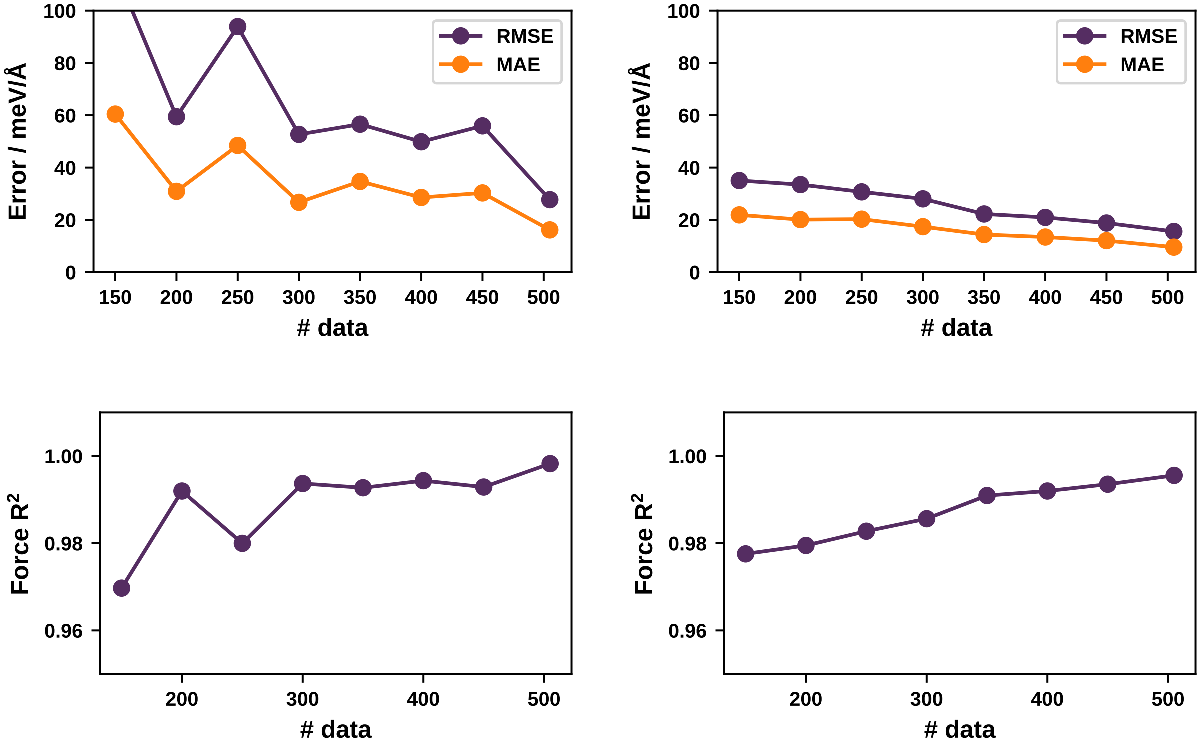

Figure S12: Learning curve for left) O-atoms; right) Cu-atoms

S3 Lifetimes from different methods

S3.1 Electron-phonon coupling lifetimes from quasi-harmonic approximation

To demonstrate the impact of the finite perturbation frequency on EPC lifetimes determined through the first-order perturbation theory and the quasi-harmonic approximation, we compare the case with zero perturbation frequency (Table S11) to that with a finite perturbation frequency (Table S12) given by the frequency of the respective vibration mode.

Table S11: Electron-phonon lifetimes using zero perturbing frequencies.

	**Lifetime / ps**			
	**FT**	**FR**	**SA**	**IS**
sharp tip	187.7	24.7	93.6	7.7
blunt tip	102.3	6.5	44.8	6.5
slab adatom	116.2	11.8	60.5	9.3
slab	63.8	4.0	13.8	3.1

Table S12: Electron-phonon lifetimes using finite perturbing frequencies.

	**Lifetime / ps**			
	**FT**	**FR**	**SA**	**IS**
sharp tip	185.9	21.2	80.0	2.8
blunt tip	99.1	6.0	39.0	3.3
slab adatom	110.8	10.8	52.9	4.9
slab	47.9	3.6	12.5	1.9

S3.2 Lifetimes from equilibrium correlation analysis

Workflow of determining lifetimes. To determine the lifetimes, we used normal mode decomposition and analysis of the velocity cross-correlation function via the Welch method (see Section *Phonon-phonon coupling* in the main manuscript and Section S5 in the Supporting Information). For each system, a single long-time MD/MDEF trajectory is computed. The velocity time series contained in this trajectory is then projected onto the vibrational eigenvectors of the system (normal-mode decomposition). To ensure numerical stability in the frequency-domain analysis, we employed the Welch method: Each projected velocity time series was divided into four segments of equal length with 50 % overlap. A cosine-shaped window function was applied to each segment to taper the edges smoothly to zero, thereby reducing spectral leakage and numerical noise. Cross-spectra were then computed for each segment using the formalism described in the main manuscript and subsequently averaged to further suppress numerical noise. Lifetimes were extracted by fitting the averaged power spectral component of the cross-spectrum with a Lorentzian function. Lifetimes were not determined from individual, unaveraged cross-spectra, as fitting instability occasionally prevented reliable convergence of the Lorentzian profile. Figure S13 illustrates the workflow.

Convergence of the length of molecular dynamics trajectories. The length of the trajectory determines the frequency resolution of the cross-spectrum, with longer trajectories yielding finer resolution. Therefore, it is essential to ensure convergence with respect to trajectory length. Since variations in trajectory length primarily affect the resolution and quality of the frequency-domain representation, it

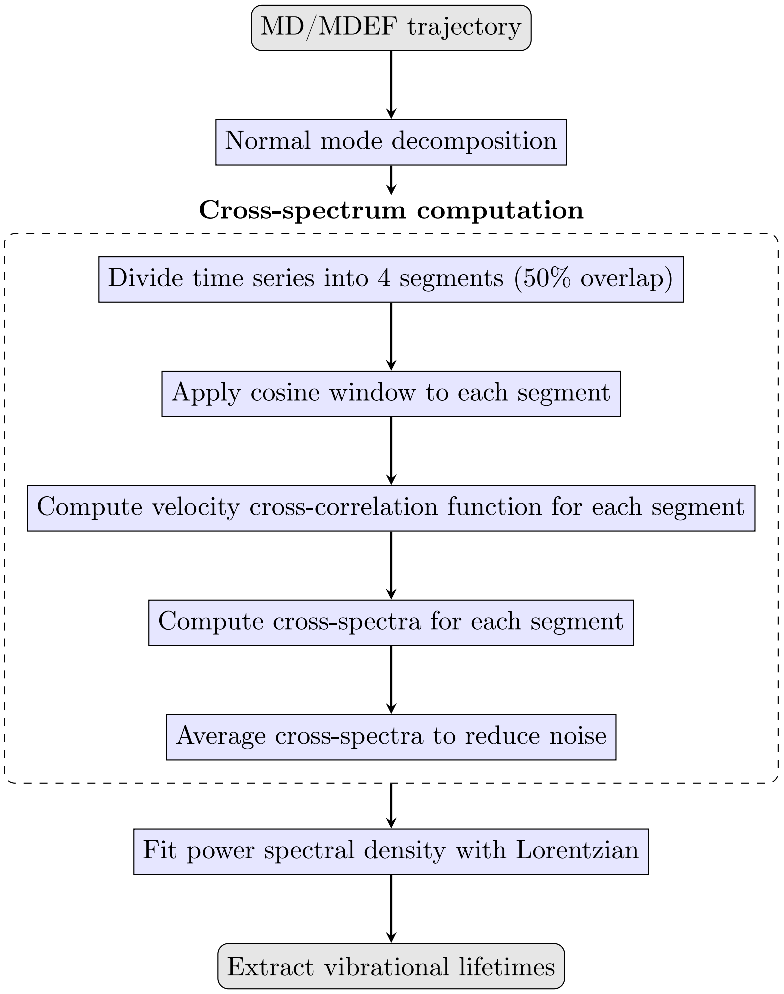

Figure S13: Workflow for determining vibrational lifetimes via equilibrium correlation analysis.

constitutes the main source of uncertainty in the computed lifetimes. We converge the lifetimes of the CO-vibration modes—frustrated translation (FT), frustrated rotation (FR), surface adsorbate stretch (SA), and internal stretch (IS)—with respect to the length of the MD-trajectories. We consider two cases: (A) regular molecular dynamics, where the lifetime is a result of phonon-phonon coupling (PPC), and (B) non-adiabatic molecular dynamics, where the lifetime is a result of EPC and PPC. Figure S14 shows the convergence for the different adsorption environments and vibration modes for the case where we have only PPC. To assess convergence, we examine how the computed lifetimes vary with different lengths extracted from a single trajectory. As a quantitative measure, we use the standard deviation of the lifetimes obtained from three progressively longer segments of the trajectory. We find that a trajectory length of approximately 2000 ps is sufficient to achieve convergence, with the associated uncertainty falling below 15 %. We note that the convergence for the FT mode of the slab shows a larger standard deviation on the final three lengths. We consider this result converged since we do not see an increase in lifetime when longer trajectories are used.

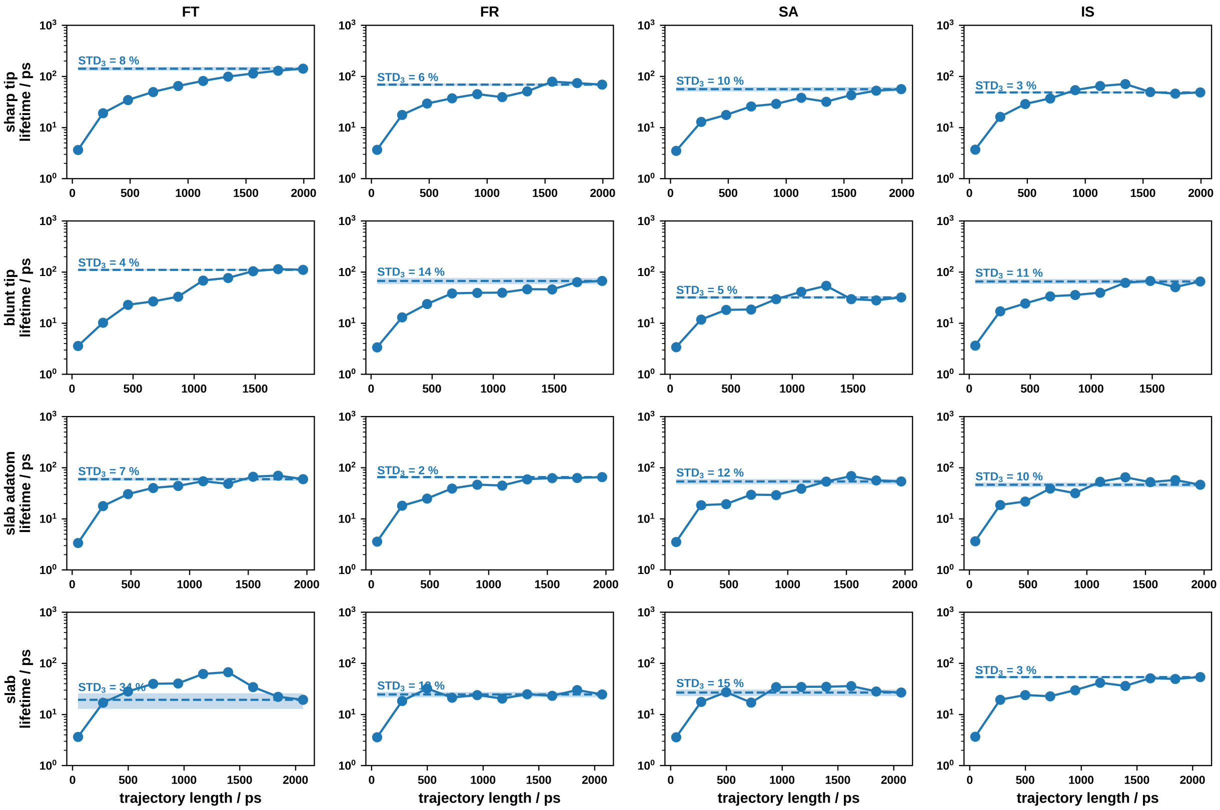

Figure S14: Convergence of CO vibrational lifetimes with respect to trajectory length; Lifetime resulting from EPC and PPC.

For the case where PPC and EPC affect the lifetimes—calculated with molecular dynamics with electronic friction (MDEF)—the convergence for the different adsorption environments and vibration modes is shown in Figure S15. Due to the increased dampening due to EPC, vibrational lifetimes are shorter than in the case where only PPC is active. This leads to better convergence. The standard deviation of the lifetime for

the final three trajectory lengths shows that a length of approximately 900 ps converges the lifetime to an uncertainty of below 50 %.

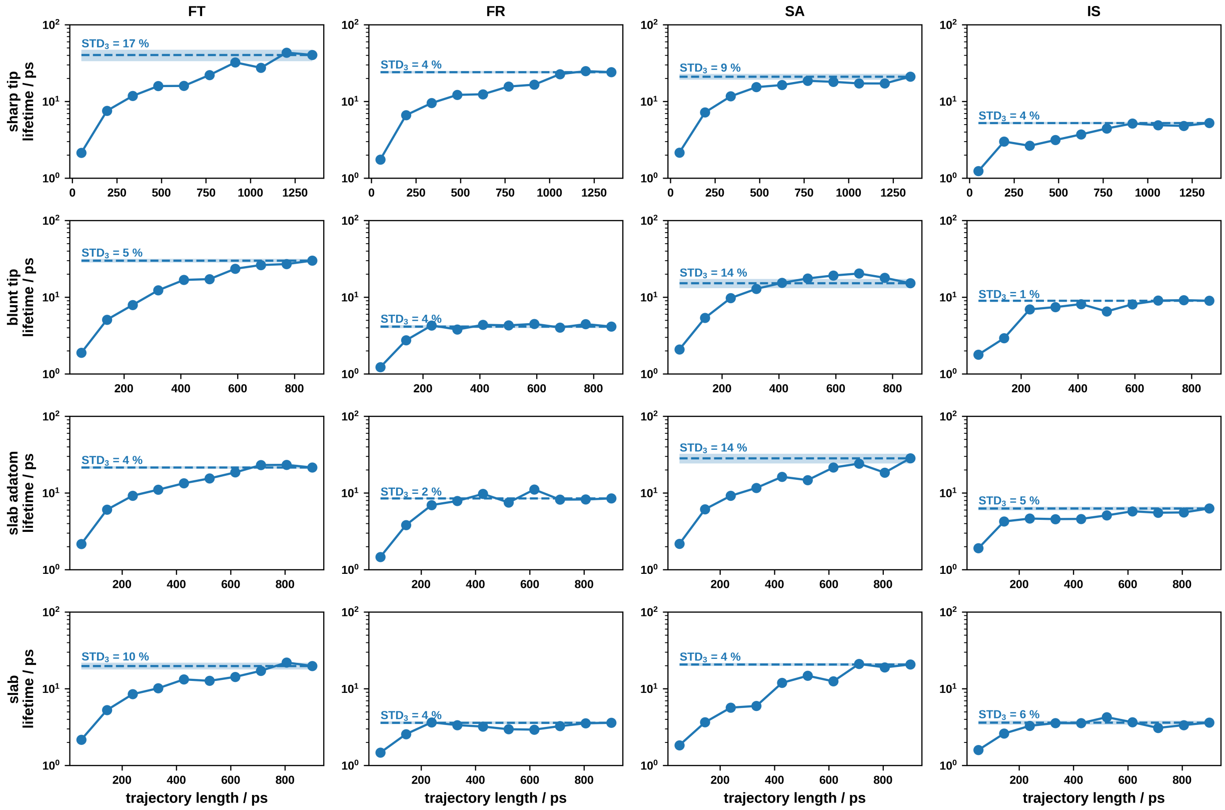

Figure S15: Convergence of CO vibrational lifetimes with respect to trajectory length; Lifetime resulting from phononic coupling.

The final values of the convergence curves for trajectory length in Figures S14 and S15 are taken as the lifetime, and the standard deviation over the last three convergence steps is taken as the uncertainty. Lifetimes determined via equilibrium correlation analysis are shown in Chapter S3.5.

Examples of the power-spectrum and the cross-spectrum. As we explain in the results chapter on PPC in the main manuscript, we determine lifetimes based on equilibrium dynamics by analyzing the cross-spectrum. This is the Fourier transform of the velocity cross-correlation function. The cross-spectrum $\tilde{C}_{\mathrm{ij}}(\omega)$ represents the coupling between the vibration modes i and j. Its diagonal elements $\tilde{C}_{\mathrm{ii}}(\omega)$ contain one peak at the characteristic frequency of the mode i, whose width corresponds to the lifetime. The diagonal elements of the cross-spectrum are called the power spectrum and are exemplarily shown for the blunt tip in Figure S16.

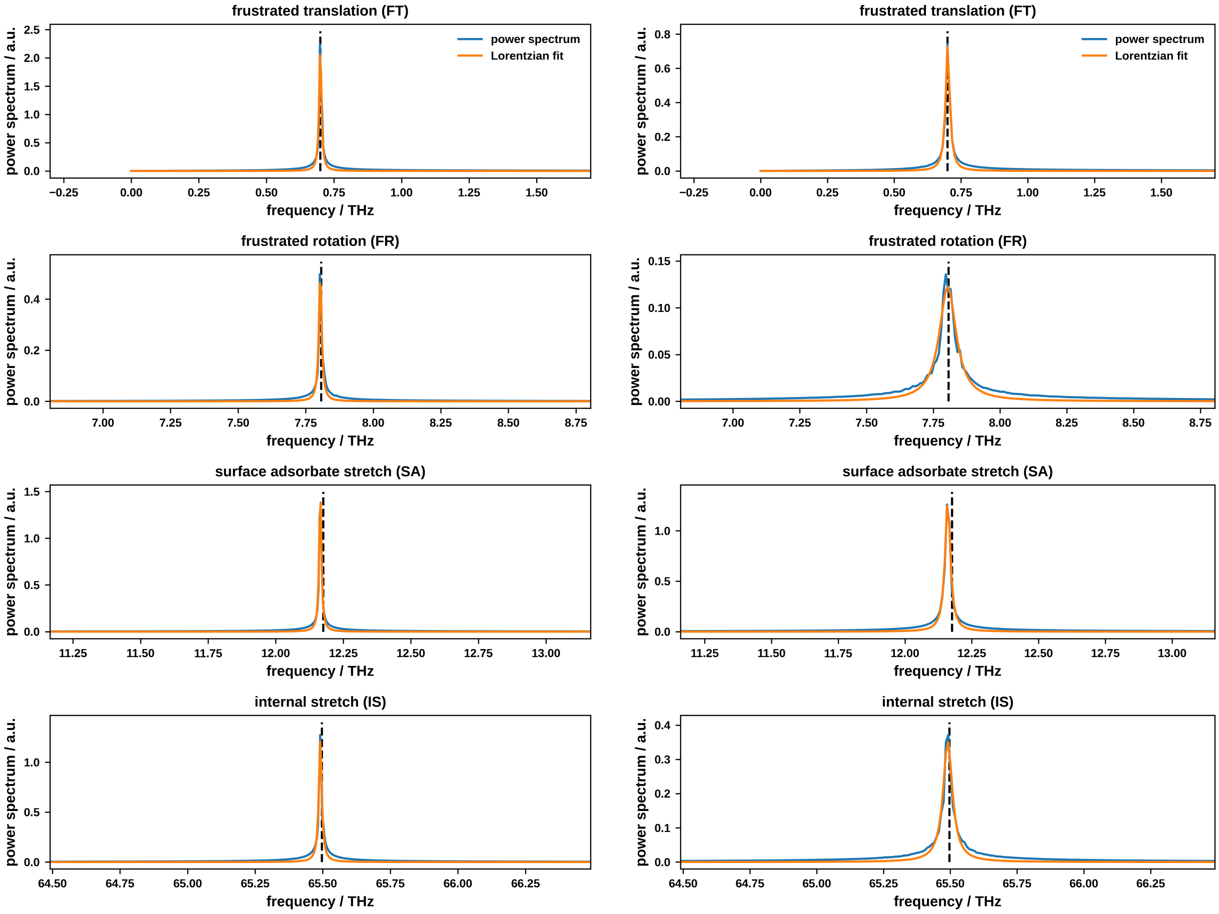

Figure S16: Power spectra of the different CO modes of the blunt tip; Left) only with PPC; Right) with EPC and PPC.

Its off-diagonal elements $\tilde{C}_{\mathrm{ij}}(\omega)$ show two peaks at the characteristic frequencies of the two vibration modes i and j. An example of the cross-spectrum between the FT and the RF mode of the CO molecule is shown in Figure S17. We note that in our work, the cross-spectrum contains coupling between CO and all metal modes.

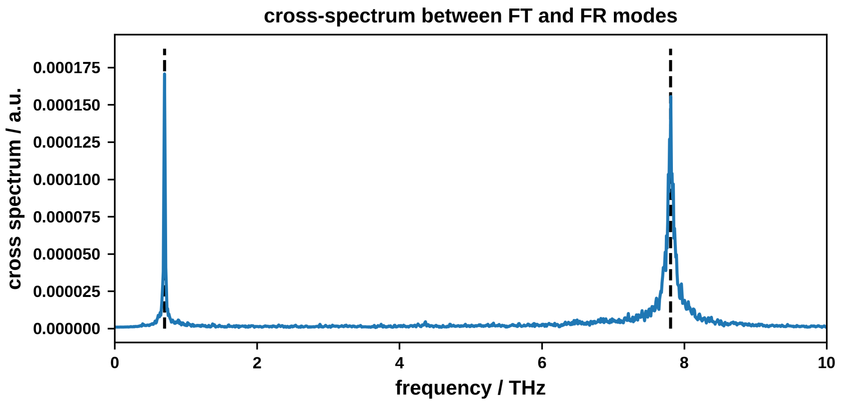

Figure S17: Cross spectrum between the FT and FR modes of the blunt tip.

S3.3 Lifetimes from kinetic energy decay

Workflow of determining lifetimes. To determine the production lifetime from explicit dynamic simulations, we conduct 64 simulations, each starting with randomised initial velocities at a temperature of 5 K. The system is then thermalised at 5 K for 50,000 MD(EF) steps. As in all dynamic simulations in this work, we use a time step of 0.1 fs. After thermalisation, we activate the CO mode of interest with 50 meV and monitor the decay of the kinetic energy envelope. To determine the kinetic energy of a given vibration mode, we use normal mode decomposition. The envelope of the kinetic energy is then fitted with an exponential function to extract the lifetime. Performing this analysis over 64 trajectories enables us to determine robust lifetimes, which are then compared to lifetimes obtained from other methods in Chapter S3.5. Figure S19 shows the workflow.

Figure S18 shows the time decay of the kinetic energy envelope following excitation of each mode with 50 meV. The decay exhibits a clear exponential behavior, consistent with the motion of a damped harmonic oscillator.

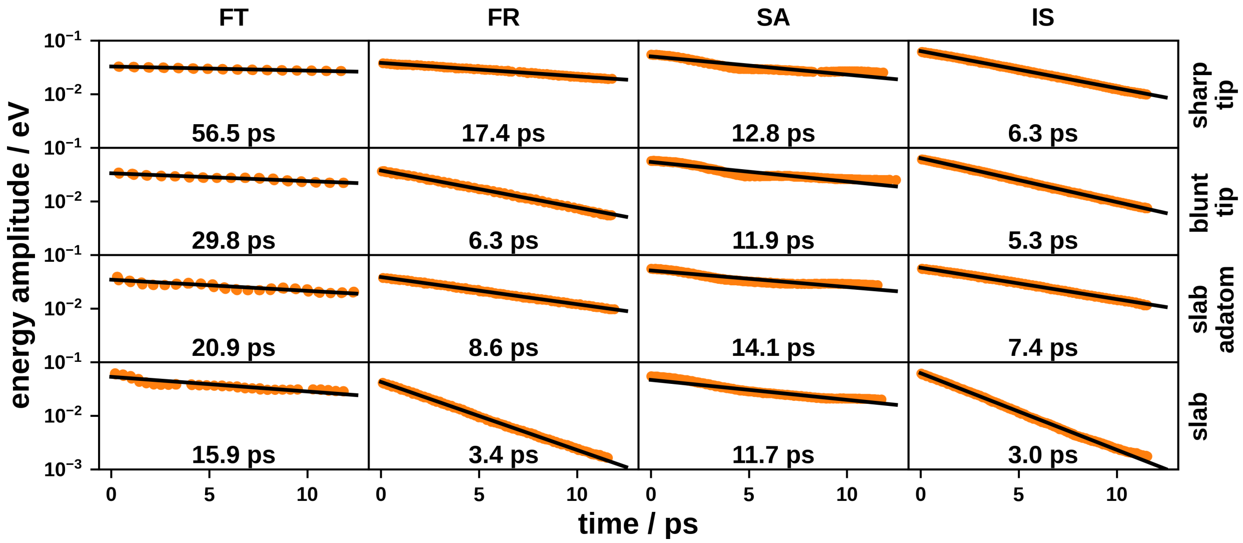

Figure S18: Time decay of the kinetic energy envelope of all adsorbate vibrations when energising each mode with 50 meV; Orange points represent the simulation data ensemble averaged over 64 trajectories; Black lines represent an exponential decay fitted to the simulation data.

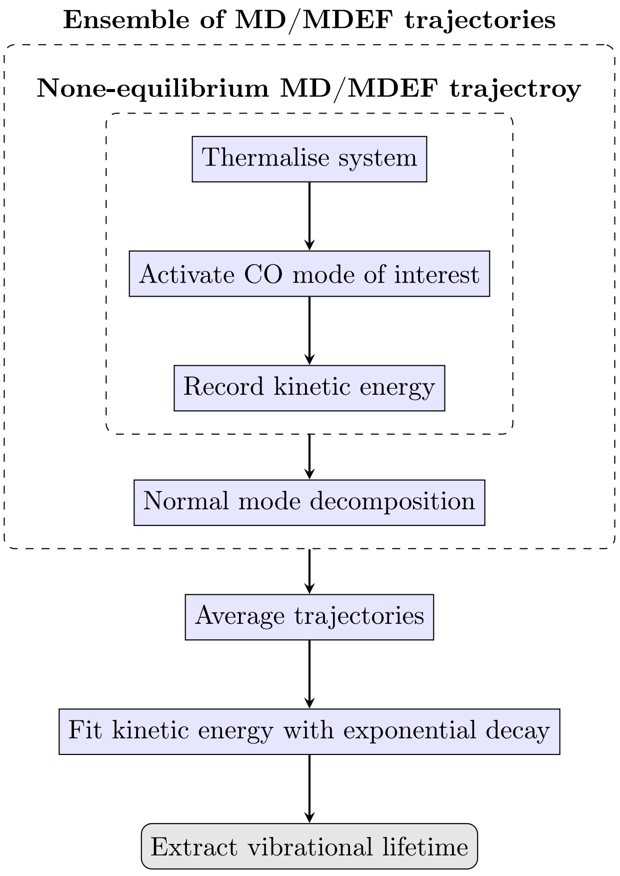

Figure S19: Workflow for determining vibrational lifetimes from explicit dynamic simulations.

Analysis of the method. We analyze the effect of activation energy by exciting the FT mode with 50 meV, 100 meV, and 200 meV. The results are shown in Figure S20. The FT mode exhibits harmonic behavior at 50 meV and remains harmonic at even higher energies when influenced by a sharp tip or a slab. Since 50 meV is large compared to the vibrational energy of the FT mode (approximately 5 meV), increasing the activation energy introduces significant anharmonicity-related effects on the lifetimes. This effect is most pronounced for the blunt tip and the slab-adatom, where a large activation energy leads to non-harmonic behavior. In contrast, for the FT mode of the sharp tip and the slab, lifetimes remain robust against changes in activation energy.

Due to anharmonicity, other modes are also activated, with the largest secondary activation occurring in the IS mode. The lifetimes of the IS mode are generally stable across different activation energies, except for very small activation energies, as observed for the IS mode of the slab. The SA mode also experiences significant secondary activation and exhibits a robust lifetime. On the slab, however, the secondary activation energy is too low, causing the decay to be lost in thermal noise and the inherent uncertainty of the simulations. The FR mode receives only minor secondary activation, with only the sharp tip providing sufficient energy to determine lifetimes. Overall, we find that the activation energy must strike a balance: it should be large enough to produce a measurable decay but small enough to minimize anharmonicity effects. Our results indicate that 50 meV achieves this balance.

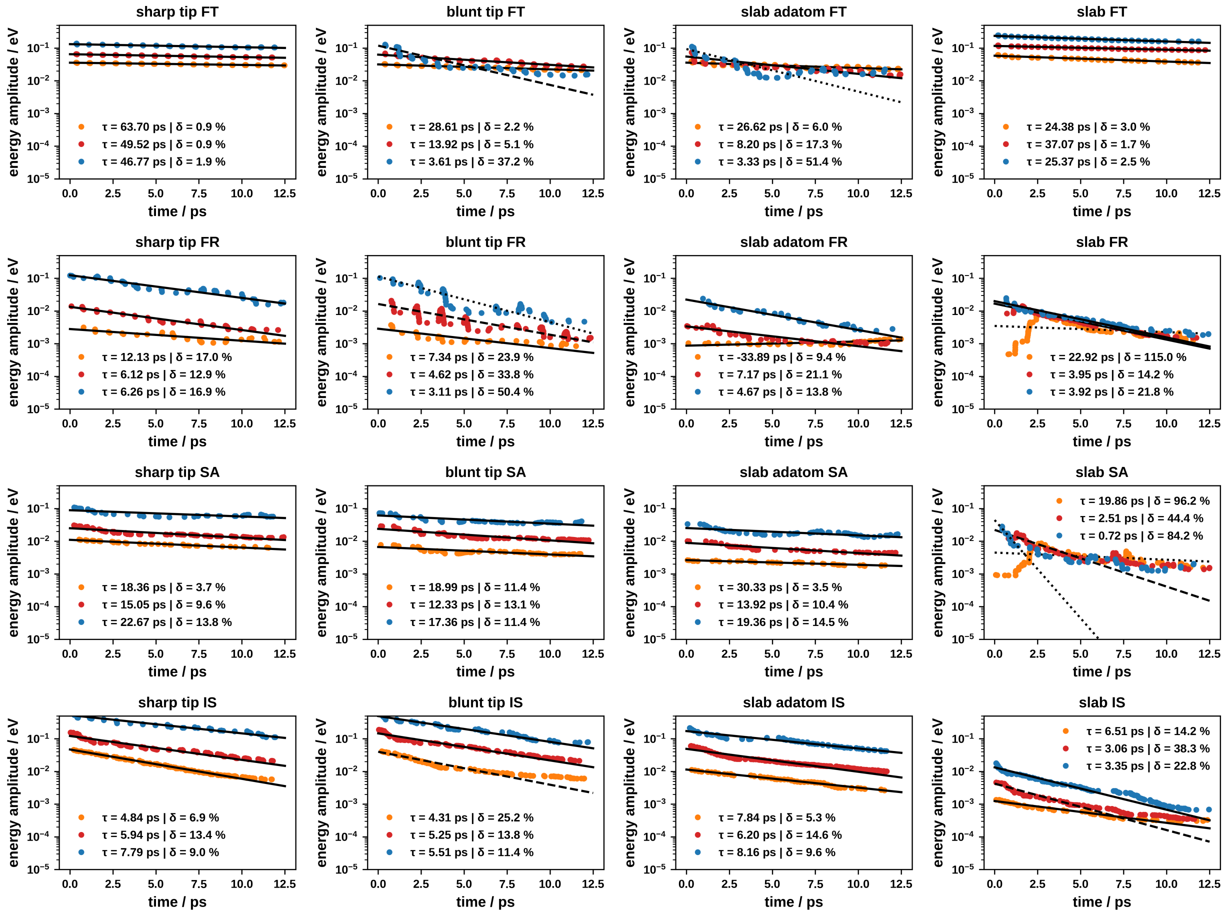

Figure S20: Energy dissipation of the four CO-modes, when the FT mode is activated with 50 meV (orange), 100 meV (red), and 200 meV (blue) of energy.

The analysis of the secondary activation of other CO modes in Figure S20 provides insight into the anharmonicity of different systems. Secondary activation energies, which serve as an indicator of anharmonicity, decrease as the substrate geometry becomes flatter. A large secondary activation indicates strong anharmonicity. Therefore, we conclude that the sharp tip is the most anharmonic system, while the slab is the most harmonic.

S3.4 Lifetimes from additive relaxation rates

Workflow of determining lifetimes. Lifetimes are obtained by adding up relaxation rates that were separately obtained for EPC and PPC. Figure S21 shows the workflow.

- **EPC.** These relaxation rates are determined in the quasi-static limit using first-order time-dependent perturbation theory. I.e., the relaxation rates are extracted from the electronic friction tensors (see Section S1.7.
- **PPC.** These relaxation rates are determined using the method of equilibrium correlation analysis on an MD trajectory (see Section S3.2).

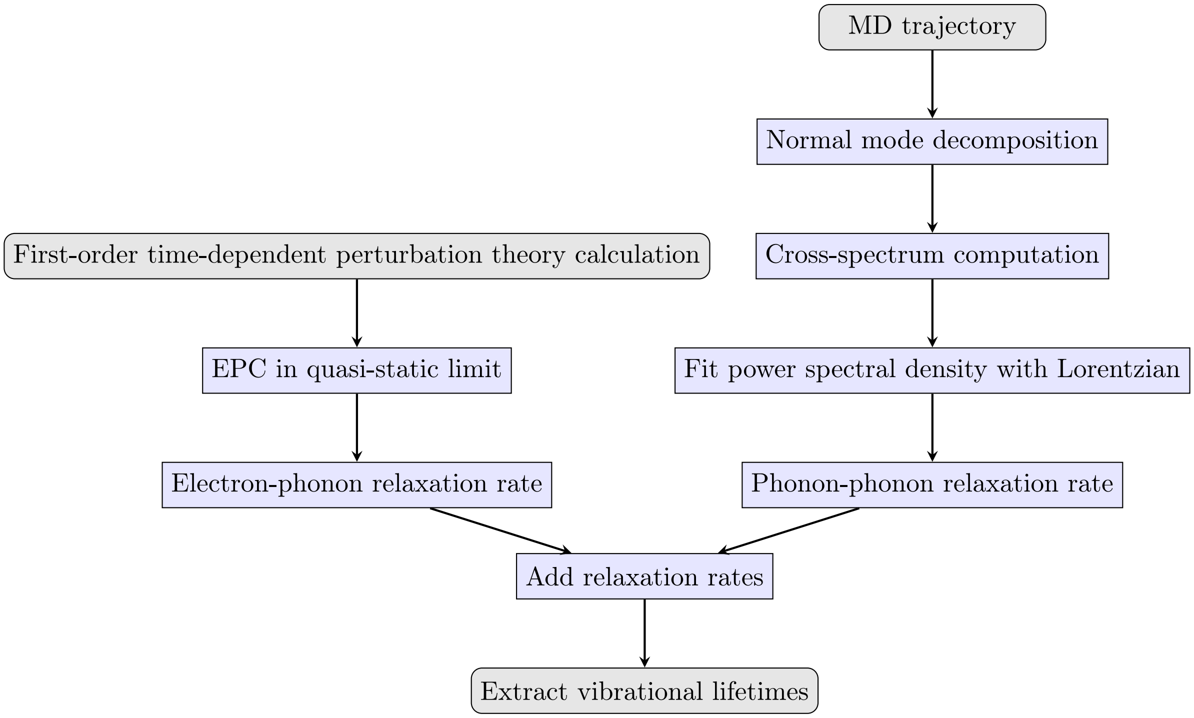

Figure S21: Workflow for determining vibrational lifetimes via additive relaxation rates.

S3.5 Comparison of lifetimes from different methods

Phonon-phonon lifetimes are calculated in two different ways (see Table S13). We attribute differences in these values to the anharmonicity of the systems, which affects the kinetic energy decay method more strongly than the equilibrium correlation analysis:

- Equilibrium correlation analysis
- Kinetic energy decay

Table S13: Comparison of lifetimes from PPC.

	equilibrium correlation analysis Lifetime / ps				kinetic energy decay Lifetime / ps			
	**FT**	**FR**	**SA**	**IS**	**FT**	**FR**	**SA**	**IS**
sharp tip	142.1	69.0	56.2	48.5	138.1	20.4	14.6	17.0
blunt tip	110.9	67.0	31.9	65.4	36.4	19.2	13.3	15.5
slab adatom	59.8	65.3	53.7	46.3	25.5	14.3	15.7	21.1
slab	19.3	24.6	26.8	53.7	21.7	8.8	21.3	15.6

Electron-phonon lifetimes are calculated in two different ways (see Table S14):

- Quasi-harmonic approximation
- Kinetic energy decay

Table S14: Comparison of lifetimes from EPC.

	quasi-harmonic approx. Lifetime / ps				kinetic energy decay Lifetime / ps			
	**FT**	**FR**	**SA**	**IS**	**FT**	**FR**	**SA**	**IS**
sharp tip	187.7	24.7	93.6	7.7	205.5	37.4	78.3	11.1
blunt tip	102.3	6.5	44.8	6.5	185.4	12.9	48.4	9.3
slab adatom	116.2	11.8	60.5	9.3	159.4	17.1	59.2	14.9
slab	63.8	4.0	13.8	3.1	105.0	7.4	19.5	6.0

Total lifetimes—including effects from EPC and PPC—are calculated with three different methods (see Table S15):

- Additive relaxation rates: Electron-phonon relaxation rate from quasi-harmonic approximation + phonon-phonon relaxation rate from equilibrium correlation analysis
- Equilibrium correlation analysis
- Kinetic energy decay

Table S15: Comparison of total lifetimes.

	additive relaxation rates Lifetime / ps				equilibrium correlation ana. Lifetime / ps				kinetic energy decay Lifetime / ps			
	**FT**	**FR**	**SA**	**IS**	**FT**	**FR**	**SA**	**IS**	**FT**	**FR**	**SA**	**IS**
sharp tip	80.9	18.2	35.1	6.7	40.4	24.1	21.1	5.2	56.5	17.4	12.8	6.3
blunt tip	53.2	5.9	18.6	5.9	30.0	4.1	15.2	9.0	29.8	6.3	11.9	5.3
slab adatom	39.5	10.0	28.5	7.7	21.5	8.5	28.4	6.3	20.9	8.6	14.1	7.4
slab	14.8	3.4	9.1	3.0	19.8	3.6	20.7	3.6	15.9	3.4	11.7	3.0

S3.6 Contributions to the relaxation rates

Figure S22 shows the contributions of EPC and EPP to the total relaxation rate (inverse lifetime). To disentangle them, we use the kinetic energy decay method, as described in the main manuscript. The contributions of PPC and EPC are relatively independent of the adsorption environment, but show a strong dependence on the vibration mode.

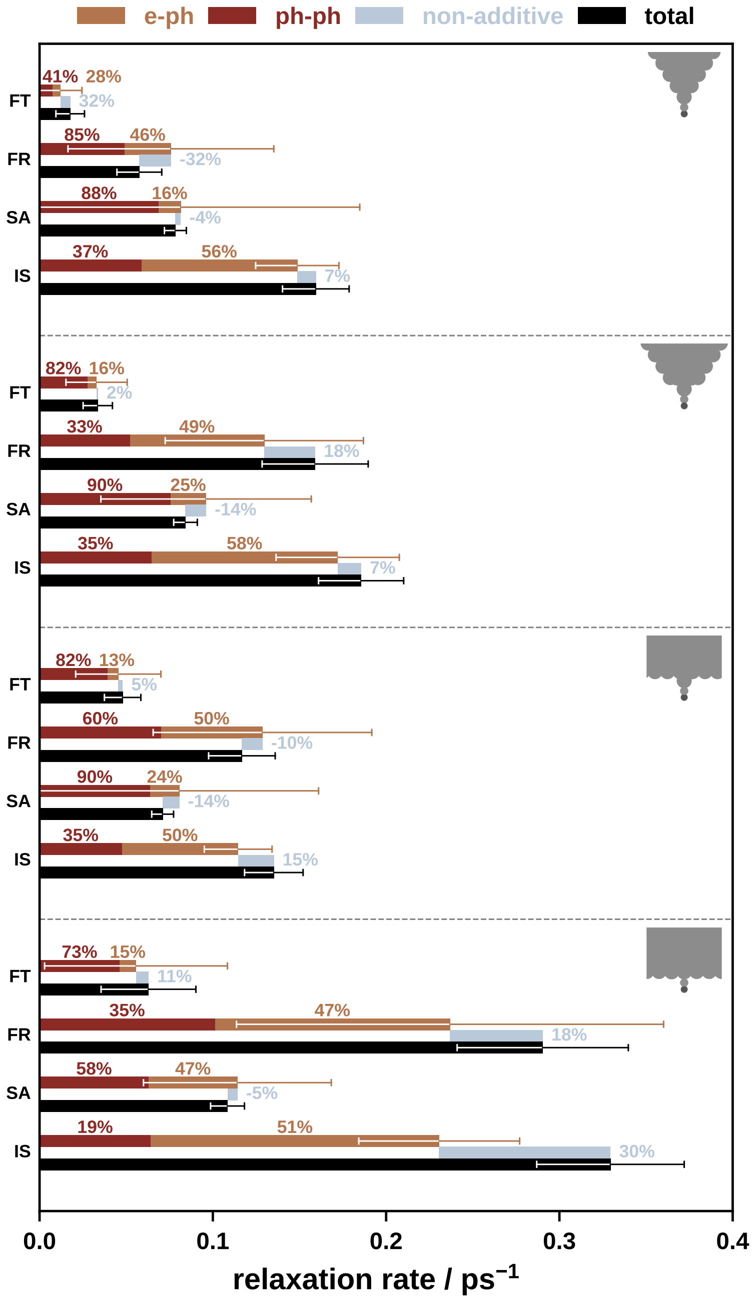

Figure S22: Contributions to relaxation rates by EPC and PPC when determined using the kinetic energy decay method: Light brown: electron-phonon relaxation rate extracted from MDEF; dark-brown: phonon-phonon relaxation rate determined with MD; slate blue: non-additive component of the relaxation rate; black: total relaxation rate from MDEF.

S4 Comparison to other coinage metals

The vibrational relaxation rate experienced by an adsorbate on a surface is governed by the coupling to both substrate phonons and electronic excitations. Here, we will offer a qualitative discussion of the expected coupling strengths of CO on different coinage metals.

S4.1 Vibrational properties and phonon-phonon coupling

The vibrational coupling strength between an adsorbate and a surface arises from several factors: Most fundamentally, it depends on the strength of the interaction between the adsorbate and substrate, which governs the degree of vibrational mode hybridization, and on the mass mismatch between the two, which determines the extent of spectral overlap between molecular vibrations and substrate phonons. However, other factors also play a role. These include the phonon density of states of the substrate, which reflects both its atomic mass and bonding characteristics; the adsorption sites; and the electronic structure of the surface, particularly the extent of orbital hybridization, which can introduce anharmonicities that modulate coupling strength.

Table S16: Experimental vibrational energies of a CO molecule on 111-surfaces of coinage metals.

	vibrational energies / meV			
	**FT**	**FR**	**SA**	**IS**
Cu(111)[8,9]	4.07	36.47	41.41	257.63
Ag(111)[11]	-	-	-	265.94
Ag nanoparticles[12]	-	7.94	19.84	261.98
Au(111)[13]	-	-	-	263.34

Binding energies of CO on Ag and Au[13,14] are smaller than on Cu. This weaker interaction leads to a reduced hybridization between molecular and surface vibrational modes, which manifests as an increase in the IS vibrational energies and a corresponding decrease in the FT, FR, and SA vibrational energies, as shown in Table S16.

The phonon density of states of the substrates shifts to lower energies from Cu to Ag to Au (Figure S23), as a result of the increasing atomic mass and weaker bonding in the lattice. This shift reduces the vibrational spectral overlap between the IS mode of CO and the substrate, leading to a more pronounced decoupling of the IS mode on Ag and Au compared to Cu. In contrast, the FT, FR, and SA modes of CO on Ag and Au fall within the low-energy phonon range of the substrate and therefore remain more effectively coupled.

Overall, the larger mass mismatch between CO and Ag or Au, compared to CO and Cu, is expected to reduce PPC, particularly for high-frequency modes such as the internal stretch. As a result, vibrational energy relaxation rates via phonon–phonon pathways are likely to be lower for CO adsorbed on Ag and Au surfaces than on Cu. Given the chemical similarity of the coinage metals—all of which are predominantly s-band conductors—this trend is expected to persist even in more complex geometries such as rough surfaces and nanoscale tips.

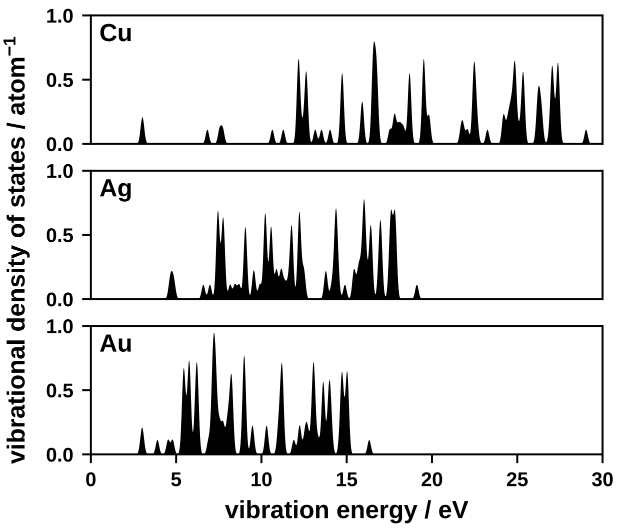

Figure S23: Comparison of vibrational density of states of coinage metal surfaces.

S4.2 Electron-phonon coupling

The efficiency of EPC depends on the local electronic structure, including the density of states near the Fermi level, the degree of adsorbate–metal orbital hybridization, and the symmetry of the vibrational mode, as we have shown in the main manuscript.

Table S17: Comparison of calculated lifetimes.[15]

	**Adsorption height / Å**	**Lifetime / ps**			
		**FT**	**FR**	**SA**	**IS**
Cu(111)	1.9	-	1.7–2.6	-	3.8–4.9
Au(111) physisorbed	4.0	-	6–17	-	230–500
Au(111) chemisorbed	2.0	-	2.5-4.2	-	2.8-3.8

Coinage metals such as Cu, Ag, and Au are electronically similar due to their filled d-bands and s-band conduction. Given similar adsorption conditions, one would expect the lifetimes of CO on Ag and Au to be comparable to those on Cu. However, CO interacts weakly with Ag and Au. For instance, on Au(111), the reported adsorption height of CO is 4.0 Å, consistent with physisorption, whereas on Cu(111) it is only 1.9 Å, indicating chemisorption.[15] The larger adsorption distance on Au reduces the efficiency of EPC. Lifetimes of the IS mode measured for CO on Au(111) are on the order of 100 ps.[16] This result is reproduced by simulations, which also predict a significantly larger lifetime of the FR mode on Au compared to Cu.[15] Notably, if simulations assume that the adsorption height of CO on Au is comparable to that on Cu, lifetimes match (see Table S17). A similar trend is observed for Ag(111): Simulations assumed a chemisorbed adsorption geometry, yielding a lifetime of the IS mode comparable in magnitude to that on Cu.[17]

The binding energies of CO on Au become significantly stronger on low coordination sites, such as step edges.[13] Moreover, vibration frequencies of the IS mode are decreased if CO is adsorbed on an adatom,[18] indicating a stronger hybridisation between CO and the Au surface. Consequently, we expect EPC to be enhanced for CO adsorbed at low-coordination sites on Au. However, as on Cu, the local density of electronic

states available for excitations tends to decrease with reduced atomic coordination, which may counteract the increase in hybridization and partially limit the overall enhancement in coupling strength.

In summary, on a flat surface, EPC on coinage metals is similar, given a similar adsorption height. However, due to the weaker binding of CO on Au (and likely on Ag), the adsorption height increases, leading to a substantial reduction in EPC. Despite this, we anticipate that the trends in mode-specificity observed on Cu persist on Ag and Au, given the electronic similarity of coinage metals. In the case of CO adsorption on adatoms or tip-like geometries, we expect that qualitative trends predicted for Cu persist across the other coinage metals. However, on surfaces with lower atomic coordination, the enhanced molecule–substrate interaction may result in shorter vibrational lifetimes on Ag and Au relative to the flat surface.

S5 Analysis of the velocity cross-correlation function

In the ideal case, the velocity autocorrelation function has the following form:

$$y(t) = \cos(2\pi f t)e^{-t/\tau}, t \geq 0 \tag{2}$$

Here f is the frequency of the underlying vibration mode and τ is its lifetime. We can determine the lifetime by analysing the Fourier transform of $y(t)$

$$Y(\nu) = \int_0^\infty y(t)\, e^{-i2\pi\nu t}\, dt \tag{3}$$

Substituting $y(t) = \cos(2\pi f t)\, e^{-t/\tau}$:

$$Y(\nu) = \int_0^\infty \cos(2\pi f t)\, e^{-t/\tau}\, e^{-i2\pi\nu t}\, dt \tag{4}$$

Using Euler's formula to express $\cos(2\pi f t)$:

$$Y(\nu) = \int_0^\infty \frac{1}{2}\left(e^{i2\pi f t} + e^{-i2\pi f t}\right) e^{-t/\tau}\, e^{-i2\pi\nu t}\, dt \tag{5}$$

Expanding:

$$Y(\nu) = \frac{1}{2}\int_0^\infty e^{t\left(-\frac{1}{\tau}+i(2\pi f-2\pi\nu)\right)}\, dt + \frac{1}{2}\int_0^\infty e^{t\left(-\frac{1}{\tau}-i(2\pi f+2\pi\nu)\right)}\, dt \tag{6}$$

Each term can be integrated separately. For the first term:

$$\int_0^\infty e^{t\left(-\frac{1}{\tau}+i(2\pi f-2\pi\nu)\right)}\, dt = \frac{1}{\frac{1}{\tau} - i(2\pi f - 2\pi\nu)} \tag{7}$$

And for the second term:

$$\int_0^\infty e^{t\left(-\frac{1}{\tau}-i(2\pi f+2\pi\nu)\right)}\, dt = \frac{1}{\frac{1}{\tau} + i(2\pi f + 2\pi\nu)} \tag{8}$$

Thus, the full expression for $Y(\nu)$ is:

$$Y(\nu) = \frac{1}{2}\left(\frac{1}{\frac{1}{\tau} - i(2\pi f - 2\pi\nu)} + \frac{1}{\frac{1}{\tau} + i(2\pi f + 2\pi\nu)}\right) \tag{9}$$

To separate real and imaginary parts:

$$\mathrm{Re}(Y(\nu)) = \frac{1}{2}\left(\frac{\frac{1}{\tau}}{\left(\frac{1}{\tau}\right)^2 + (2\pi f - 2\pi\nu)^2} + \frac{\frac{1}{\tau}}{\left(\frac{1}{\tau}\right)^2 + (2\pi f + 2\pi\nu)^2}\right) \tag{10}$$

$$\mathrm{Im}(Y(\nu)) = \frac{1}{2}\left(\frac{(2\pi f - 2\pi\nu)}{\left(\frac{1}{\tau}\right)^2 + (2\pi f - 2\pi\nu)^2} - \frac{(2\pi f + 2\pi\nu)}{\left(\frac{1}{\tau}\right)^2 + (2\pi f + 2\pi\nu)^2}\right) \tag{11}$$

To determine the lifetime, we look at the real positive part of the power spectrum.

$$\mathrm{Re}(Y(\nu+)) = \frac{1}{2}\frac{\frac{1}{\tau}}{\left(\frac{1}{\tau}\right)^2 + (2\pi f - 2\pi\nu)^2} \tag{12}$$

$$= \frac{1}{2}\frac{\frac{1}{\tau}}{\left(\frac{1}{\tau}\right)^2 [1 + (2\pi\tau)^2(f-\nu)^2]} \tag{13}$$

$$= \frac{1}{2}\frac{1}{\frac{1}{\tau}[1 + (2\pi\tau)^2(f-\nu)^2]} \tag{14}$$

With $\gamma = 1/2\pi\tau$ we get a Lorentzian.

$$\mathrm{Re}(Y(\nu+)) = \frac{1}{4\pi\gamma\left[1 + \left(\frac{f-\nu}{\gamma}\right)^2\right]} \tag{15}$$

S6 Coupling strength between vibrational modes

To estimate the coupling strength between vibrational modes from molecular dynamics, we analyse the time-correlated behaviour of the vibrational amplitudes. To understand our analysis, let us consider two weakly coupled vibrational modes k and l and assume that the interaction Hamiltonian between modes k and l is linear and has the following form.

$$H_{\mathrm{int}} = \lambda q_{\mathrm{k}} q_{\mathrm{l}}, \tag{16}$$

Here q_{k} and q_{l} are the displacement operators for modes k and l and λ is the coupling constant representing the interaction strength between these modes. This Hamiltonian term describes the energy contribution due to the interaction of the two vibrational modes. The coupling constant λ quantifies how strongly the energy of one mode is influenced by the displacement of the other mode. From the Hamiltonian, we can derive equations of motion for the displacements q_{k} and q_{l}. We use Hamilton's equations to find that the interaction introduces additional terms into the equations of motion of each mode that are proportional to λ.

$$\ddot{q}_{\mathrm{k}} + \omega_{\mathrm{k}}^2 q_{\mathrm{k}} = -\lambda q_{\mathrm{l}} \tag{17}$$

$$\ddot{q}_{\mathrm{l}} + \omega_{\mathrm{l}}^2 q_{\mathrm{l}} = -\lambda q_{\mathrm{k}} \tag{18}$$

Here ω_{k} and ω_{l} are the natural frequencies of modes k and l. These coupled equations of motion lead to oscillatory energy exchange between the two modes, with the rate of exchange depending on λ.

S6.1 Vibrational modes and amplitudes

Suppose we have two vibrational modes q_k and q_l with time-dependent amplitudes $A_\mathrm{k}(t)$ and $A_\mathrm{l}(t)$ obtained from an MD trajectory, often through normal mode analysis or Fourier decomposition of atomic displacements. The amplitudes $A_\mathrm{k}(t)$ contain information about the coupling. The displacement for each mode q_k can be expressed as:

$$q_\mathrm{k}(t) = A_\mathrm{k}(t)\exp(i\Omega_\mathrm{k}t + \phi_\mathrm{k}) \tag{19}$$

$$q_\mathrm{l}(t) = A_\mathrm{l}(t)\exp(i\Omega_\mathrm{l}t + \phi_\mathrm{l}) \tag{20}$$

Here ω_k and ω_l are the characteristic frequencies of modes k and l. ϕ_k and ϕ_l are the phases.
Substituting the vibrational mode solutions in Equation 18 we get.

$$\ddot{A}_\mathrm{k}(t) + \tilde{\Omega}_\mathrm{k}^2 A_k(t) + \lambda A_\mathrm{l}(t) = 0 \tag{21}$$

$$\ddot{A}_\mathrm{l}(t) + \tilde{\Omega}_\mathrm{l}^2 A_l(t) + \lambda A_\mathrm{k}(t) = 0 \tag{22}$$

Here $\tilde{\Omega}_\mathrm{k}^2 = \omega_\mathrm{k}^2 - \Omega_\mathrm{k}^2$ (same for l). To solve the system of coupled differential equations we assume solutions of the form:

$$A_\mathrm{k}(t) = A_\mathrm{k0}e^{i\omega t} \tag{23}$$

$$A_\mathrm{l}(t) = A_\mathrm{l0}e^{i\omega t}. \tag{24}$$

Substituting these into the equations, we get:

$$(-\omega^2 + \tilde{\Omega}_\mathrm{k}^2)A_\mathrm{k0} + \lambda A_\mathrm{l0} = 0, \tag{25}$$

$$\lambda A_\mathrm{k0} + (-\omega^2 + \tilde{\Omega}_\mathrm{l}^2)A_\mathrm{l0} = 0. \tag{26}$$

This can be written in matrix form:

$$\begin{pmatrix} \tilde{\Omega}_\mathrm{k}^2 - \omega^2 & \lambda \\ \lambda & \tilde{\Omega}_\mathrm{l}^2 - \omega^2 \end{pmatrix} \begin{pmatrix} A_\mathrm{k0} \\ A_\mathrm{l0} \end{pmatrix} = 0 \tag{27}$$

For non-trivial solutions, the determinant of this matrix must be zero:

$$(\tilde{\Omega}_\mathrm{k}^2 - \omega^2)(\tilde{\Omega}_\mathrm{l}^2 - \omega^2) - \lambda^2 = 0. \tag{28}$$

Expanding, we obtain:

$$\omega^4 - (\tilde{\Omega}_\mathrm{k}^2 + \tilde{\Omega}_\mathrm{l}^2)\omega^2 + \tilde{\Omega}_\mathrm{k}^2\tilde{\Omega}_\mathrm{l}^2 - \lambda^2 = 0. \tag{29}$$

This is a quadratic equation in ω^2, with solutions:

$$\omega^2 = \frac{\tilde{\Omega}_\mathrm{k}^2 + \tilde{\Omega}_\mathrm{l}^2 \pm \sqrt{(\tilde{\Omega}_\mathrm{k}^2 - \tilde{\Omega}_\mathrm{l}^2)^2 + 4\lambda^2}}{2}. \tag{30}$$

We can rewrite the eigenvalue equation 30 as follows:

$$\omega^2 = \frac{\tilde{\Omega}_\mathrm{k}^2 + \tilde{\Omega}_\mathrm{l}^2}{2} \pm \frac{1}{2}\sqrt{(\tilde{\Omega}_\mathrm{k}^2 - \tilde{\Omega}_\mathrm{l}^2)^2 + 4\lambda^2} \tag{31}$$

For $\lambda \ll |\tilde{\Omega}_\mathrm{k}^2 - \tilde{\Omega}_\mathrm{l}^2|$, we expand the square root term using the binomial expansion:

$$\sqrt{(\tilde{\Omega}_\mathrm{k}^2 - \tilde{\Omega}_\mathrm{l}^2)^2 + 4\lambda^2} = |\tilde{\Omega}_\mathrm{k}^2 - \tilde{\Omega}_\mathrm{l}^2|\sqrt{1 + \frac{2\lambda^2}{(\tilde{\Omega}_\mathrm{k}^2 - \tilde{\Omega}_\mathrm{l}^2)^2}} \approx |\tilde{\Omega}_\mathrm{k}^2 - \tilde{\Omega}_\mathrm{l}^2|\left(1 + \frac{2\lambda^2}{(\tilde{\Omega}_\mathrm{k}^2 - \tilde{\Omega}_\mathrm{l}^2)^2}\right). \tag{32}$$

Substituting this approximation into the expression for ω^2, we get for the lower eigenvalue (ω_1^2):

$$\omega_1^2 = \frac{\tilde{\Omega}_\mathrm{k}^2 + \tilde{\Omega}_\mathrm{l}^2}{2} - \frac{1}{2}\sqrt{(\tilde{\Omega}_\mathrm{k}^2 - \tilde{\Omega}_\mathrm{l}^2)^2 + 4\lambda^2}. \tag{33}$$

Using the approximation for the square root:

$$\omega_1^2 \approx \frac{\tilde{\Omega}_\mathrm{k}^2 + \tilde{\Omega}_\mathrm{l}^2}{2} - \frac{1}{2}|\tilde{\Omega}_\mathrm{k}^2 - \tilde{\Omega}_\mathrm{l}^2|\left(1 + \frac{2\lambda^2}{(\tilde{\Omega}_\mathrm{k}^2 - \tilde{\Omega}_\mathrm{l}^2)^2}\right). \tag{34}$$

Simplify:

$$\omega_1^2 \approx \min(\tilde{\Omega}_\mathrm{k}^2, \tilde{\Omega}_\mathrm{l}^2) - \frac{\lambda^2}{|\tilde{\Omega}_\mathrm{k}^2 - \tilde{\Omega}_\mathrm{l}^2|}. \tag{35}$$

For the higher eigenvalue (ω_2^2):

$$\omega_2^2 = \frac{\tilde{\Omega}_\mathrm{k}^2 + \tilde{\Omega}_\mathrm{l}^2}{2} + \frac{1}{2}\sqrt{(\tilde{\Omega}_\mathrm{k}^2 - \tilde{\Omega}_\mathrm{l}^2)^2 + 4\lambda^2}. \tag{36}$$

Using the approximation for the square root:

$$\omega_2^2 \approx \frac{\tilde{\Omega}_\mathrm{k}^2 + \tilde{\Omega}_\mathrm{l}^2}{2} + \frac{1}{2}|\tilde{\Omega}_\mathrm{k}^2 - \tilde{\Omega}_\mathrm{l}^2|\left(1 + \frac{2\lambda^2}{(\tilde{\Omega}_\mathrm{k}^2 - \tilde{\Omega}_\mathrm{l}^2)^2}\right). \tag{37}$$

Simplify:

$$\omega_2^2 \approx \max(\tilde{\Omega}_\mathrm{k}^2, \tilde{\Omega}_\mathrm{l}^2) + \frac{\lambda^2}{|\tilde{\Omega}_\mathrm{k}^2 - \tilde{\Omega}_\mathrm{l}^2|}. \tag{38}$$

To determine the eigenvectors, we can rewrite equation 27 to get:

$$(\tilde{\Omega}_\mathrm{k}^2 - \omega^2)A_{\mathrm{k}0} + \lambda A_{\mathrm{l}0} = 0 \tag{39}$$

$$\lambda A_{\mathrm{k}0} + (\tilde{\Omega}_\mathrm{l}^2 - \omega^2)A_{\mathrm{l}0} = 0 \tag{40}$$

From the first equation:

$$\frac{A_{\mathrm{k}0}}{A_{\mathrm{l}0}} = -\frac{\lambda}{\tilde{\Omega}_\mathrm{k}^2 - \omega^2}. \tag{41}$$

From the second equation:

$$\frac{A_{\mathrm{l}0}}{A_{\mathrm{k}0}} = -\frac{\lambda}{\tilde{\Omega}_\mathrm{l}^2 - \omega^2}. \tag{42}$$

These two ratios are consistent because:

$$\frac{A_{\mathrm{k0}}}{A_{\mathrm{l0}}} \cdot \frac{A_{\mathrm{l0}}}{A_{\mathrm{k0}}} = 1. \tag{43}$$

The eigenvalues are approximately:

$$\omega_1^2 \approx \min(\tilde{\Omega}_{\mathrm{k}}^2, \tilde{\Omega}_{\mathrm{l}}^2) - \frac{\lambda^2}{|\tilde{\Omega}_{\mathrm{k}}^2 - \tilde{\Omega}_{\mathrm{l}}^2|} \tag{44}$$

$$\omega_2^2 \approx \max(\tilde{\Omega}_{\mathrm{k}}^2, \tilde{\Omega}_{\mathrm{l}}^2) + \frac{\lambda^2}{|\tilde{\Omega}_{\mathrm{k}}^2 - \tilde{\Omega}_{\mathrm{l}}^2|}. \tag{45}$$

The eigenvector for ω_1^2, the lower Eigenvalue can be calculated by substituting ω_1^2 into the first equation:

$$(\tilde{\Omega}_{\mathrm{k}}^2 - \omega_1^2)A_{\mathrm{k0}} + \lambda A_{\mathrm{l0}} = 0. \tag{46}$$

For $\omega_1^2 \approx \min(\tilde{\Omega}_{\mathrm{k}}^2, \tilde{\Omega}_{\mathrm{l}}^2)$, we approximate:

$$\tilde{\Omega}_{\mathrm{k}}^2 - \omega_1^2 \approx |\tilde{\Omega}_{\mathrm{k}}^2 - \tilde{\Omega}_{\mathrm{l}}^2|. \tag{47}$$

Thus:

$$\frac{A_{\mathrm{k0}}}{A_{\mathrm{l0}}} = -\frac{\lambda}{\tilde{\Omega}_{\mathrm{k}}^2 - \omega_1^2} \approx -\frac{\lambda}{|\tilde{\Omega}_{\mathrm{k}}^2 - \tilde{\Omega}_{\mathrm{l}}^2|}. \tag{48}$$

The eigenvector is then:

$$\mathbf{v}_1 = \begin{pmatrix} 1 \\ -\frac{\lambda}{|\tilde{\Omega}_{\mathrm{k}}^2 - \tilde{\Omega}_{\mathrm{l}}^2|} \end{pmatrix}. \tag{49}$$

We can determine the eigenvector for ω_2^2, the higher eigenvalue, by substituting ω_2^2 into the first equation:

$$(\tilde{\Omega}_{\mathrm{k}}^2 - \omega_2^2)A_{\mathrm{k0}} + \lambda A_{\mathrm{l0}} = 0. \tag{50}$$

For $\omega_2^2 \approx \max(\tilde{\Omega}_{\mathrm{k}}^2, \tilde{\Omega}_{\mathrm{l}}^2)$, we approximate:

$$\tilde{\Omega}_{\mathrm{k}}^2 - \omega_2^2 \approx -|\tilde{\Omega}_{\mathrm{k}}^2 - \tilde{\Omega}_{\mathrm{l}}^2|. \tag{51}$$

Thus:

$$\frac{A_{\mathrm{k0}}}{A_{\mathrm{l0}}} = -\frac{\lambda}{\tilde{\Omega}_{\mathrm{k}}^2 - \omega_2^2} \approx \frac{\lambda}{|\tilde{\Omega}_{\mathrm{k}}^2 - \tilde{\Omega}_{\mathrm{l}}^2|}. \tag{52}$$

The eigenvector is then:

$$\mathbf{v}_2 = \begin{pmatrix} 1 \\ \frac{\lambda}{|\tilde{\Omega}_{\mathrm{k}}^2 - \tilde{\Omega}_{\mathrm{l}}^2|} \end{pmatrix}. \tag{53}$$

These solutions correspond to two normal mode frequencies, ω_1 and ω_2, which describe the coupled oscillations of A_k and A_l.

$$A_{\mathrm{k}}(t) = A_{\mathrm{k0}}e^{-i\omega_1 t} + A'_{\mathrm{k0}}e^{-i\omega_2 t} \tag{54}$$

$$A_{\mathrm{l}}(t) = A_{\mathrm{l0}}e^{-i\omega_1 t} + A'_{\mathrm{l0}}e^{-i\omega_2 t} \tag{55}$$

The influence of the coupling constant λ on the dynamics is reflected in the cross-correlation function $C_{\mathrm{kl}}(t) = \langle A_{\mathrm{k}}(t)A_{\mathrm{l}}(0)\rangle$. Since λ mixes the motion of A_{k} and A_{l}, it will affect the amplitude and phase relationships between them. The presence of λ leads to oscillations in $C_{\mathrm{kl}}(t)$ at the combined normal mode frequencies ω_1 and ω_2, showing energy exchange between the modes A_{k} and A_{l} due to coupling.

S6.2 Cross-correlation function

To obtain the coupling strength λ we calculate the Fourier transform of the cross-correlation function $C_{\mathrm{kl}}(t)$. The cross-correlation function $C_{\mathrm{kl}}(t)$ is defined as follows:

$$C_{\mathrm{kl}}(t) = \langle A_{\mathrm{k}}(t)A_{\mathrm{l}}(0)\rangle \tag{56}$$

Here $A_{\mathrm{k}}(t)$ and $A_{\mathrm{l}}(t)$ are the time-dependent amplitudes of the vibration modes, and $\langle \cdots \rangle$ denotes an average over the ensemble. The Fourier transform of the cross-correlation function $C_{\mathrm{kl}}(t)$ is given by:

$$\tilde{C}_{\mathrm{kl}}(\omega) = \int_{-\infty}^{\infty} C_{\mathrm{kl}}(t)e^{-i\omega t}dt. \tag{57}$$

We substitute the solutions for the normal modes from Equation 55 into the cross-correlation function. Note that λ influences the amplitudes A_{k0} and A_{l0}.

$$C_{\mathrm{kl}}(t) = \langle (A_{\mathrm{k0}}e^{-i\omega_1 t} + A'_{\mathrm{k0}}e^{-i\omega_2 t})(A_{\mathrm{l0}} + A'_{\mathrm{l0}})\rangle. \tag{58}$$

From the eigenvectors, we know that:

$$A_{\mathrm{k0}} \approx A_{\mathrm{l0}}\frac{\lambda}{|\tilde{\Omega}_{\mathrm{k}}^2 - \tilde{\Omega}_{\mathrm{l}}^2|} \tag{59}$$

Therefore the cross-correlation function can be approximated as follows:

$$C_{\mathrm{kl}}(t) \propto \frac{\lambda}{|\tilde{\Omega}_{\mathrm{k}}^2 - \tilde{\Omega}_{\mathrm{l}}^2|}(e^{-i\omega_1 t} + e^{-i\omega_2 t}) \tag{60}$$

When taking the Fourier transform of $C_{kl}(t)$, we find contributions from both ω_1 and ω_2. The resulting expression can be analysed in terms of the amplitudes and their relationship to the coupling strength λ. The coupling strength can be obtained from the Fourier coefficients of the cross-correlation function:

$$\tilde{C}_{\mathrm{kl}}(\omega) \propto \frac{\lambda}{\omega^2 - \omega_1^2} + \frac{\lambda}{\omega^2 - \omega_2^2}, \tag{61}$$

leading to:

$$\lambda \propto \left.\frac{\tilde{C}_{\mathrm{kl}}(\omega)}{\omega^2 - \omega_{\mathrm{i}}^2}\right|_{\omega=\omega_{\mathrm{i}}}, \quad (i = 1, 2). \tag{62}$$

This means that by analysing the peak values of the Fourier transform at the frequencies corresponding to the normal modes, you can estimate λ. The height of the peaks $\tilde{C}_{\mathrm{kl}}(\omega)|_{\omega=\omega_{1/2}}$ is related to the coupling strength.

S6.3 Coupling strength between vibration modes

We visualise the coupling density $\rho_j(\omega)$ between a given CO mode and the other modes in the system:

$$\rho_{\mathrm{j}}(\omega) = \sum_{\mathrm{i}} \tilde{C}_{\mathrm{ij}}(\omega) \tag{63}$$

A comparison of the coupling density with and without electron-phonon interaction reveals that the coupling is significantly stronger when EPC is included. We observe the strongest coupling for the FR and IS modes. The geometry of the surface significantly affects the coupling density: The sharp tip exhibits the smallest coupling, while the slab shows the largest.

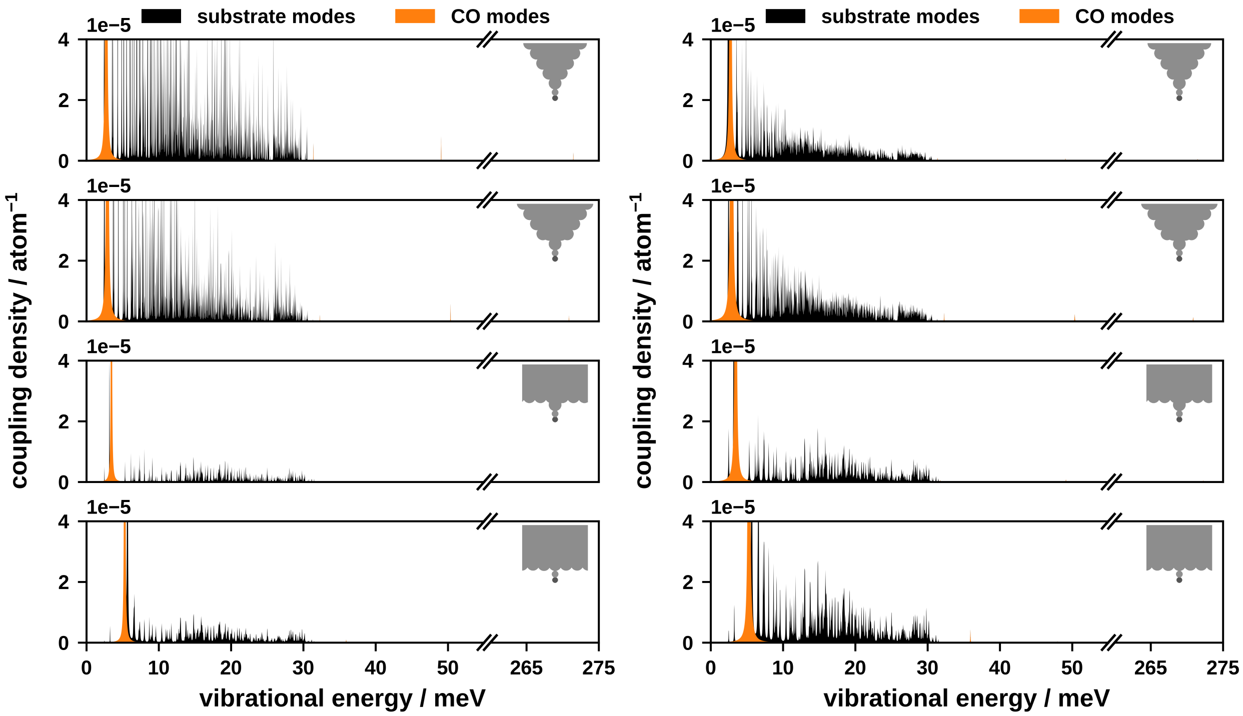

Figure S24: Coupling density of the FT mode with all other modes; Left) PPC only; Right) EPC and PPC.

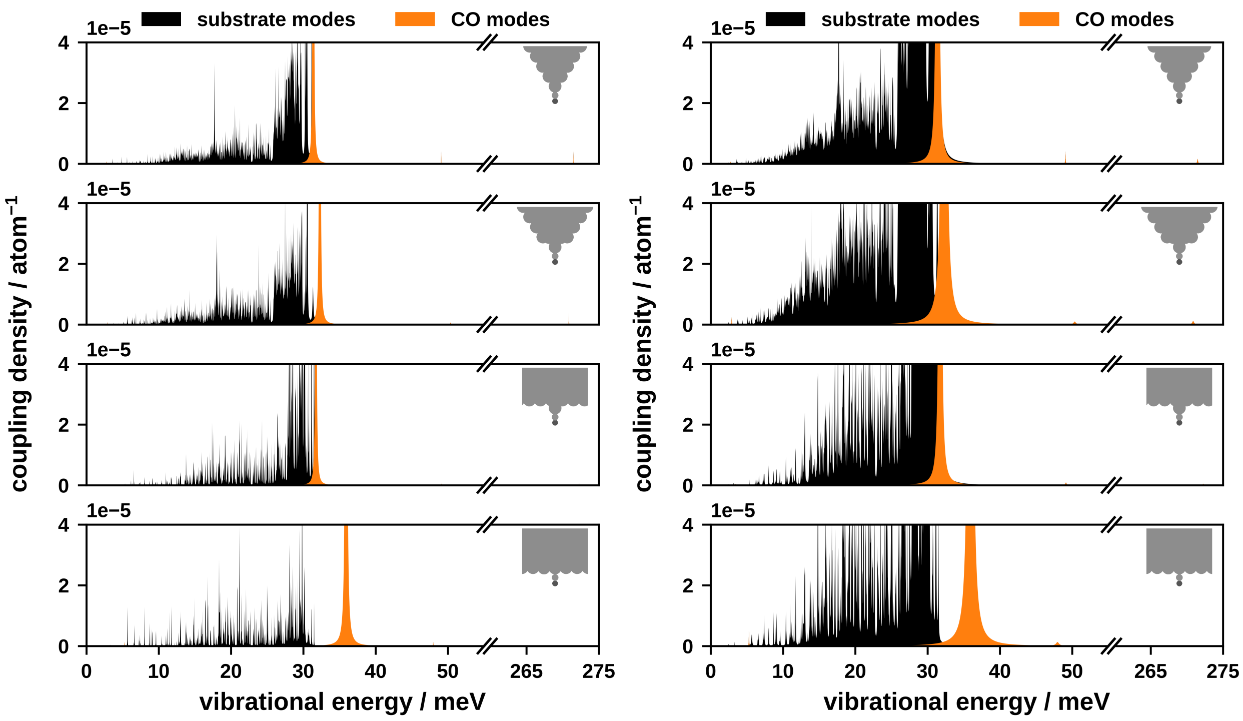

Figure S25: Coupling density of the FR mode with all other modes; Left) PPC only; Right) EPC and PPC.

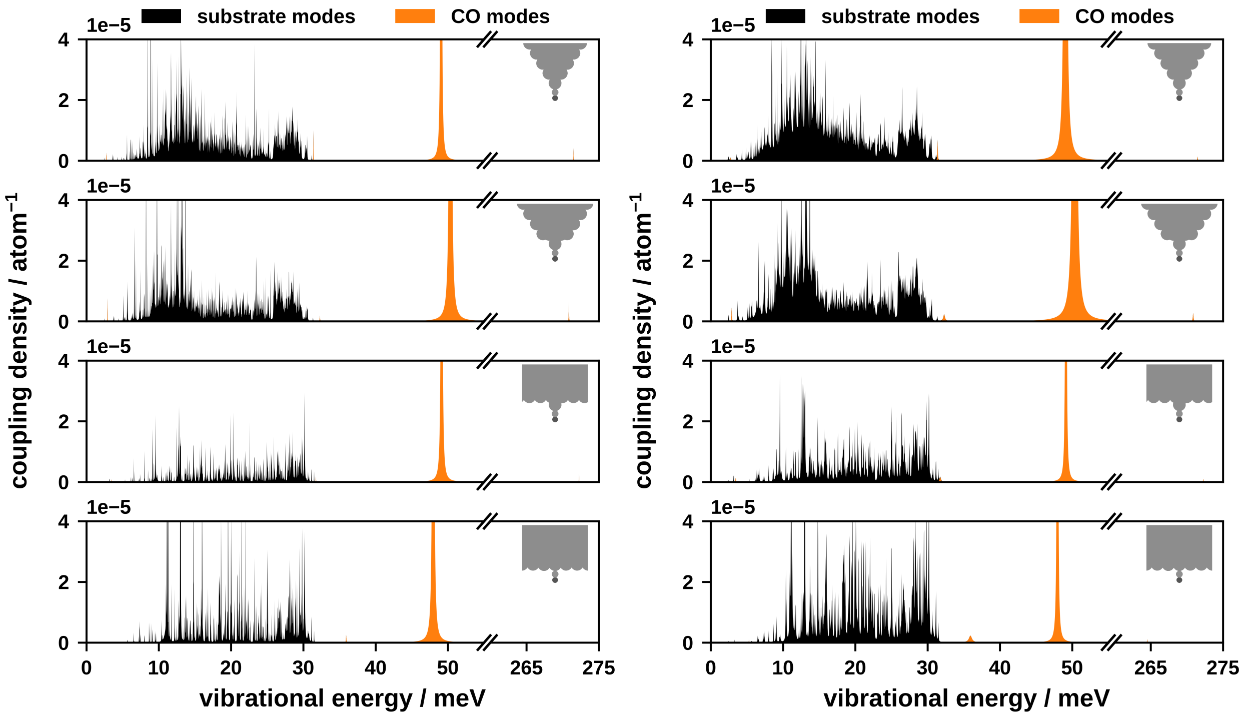

Figure S26: Coupling density of the SA mode with all other modes; Left) PPC only; Right) EPC and PPC.

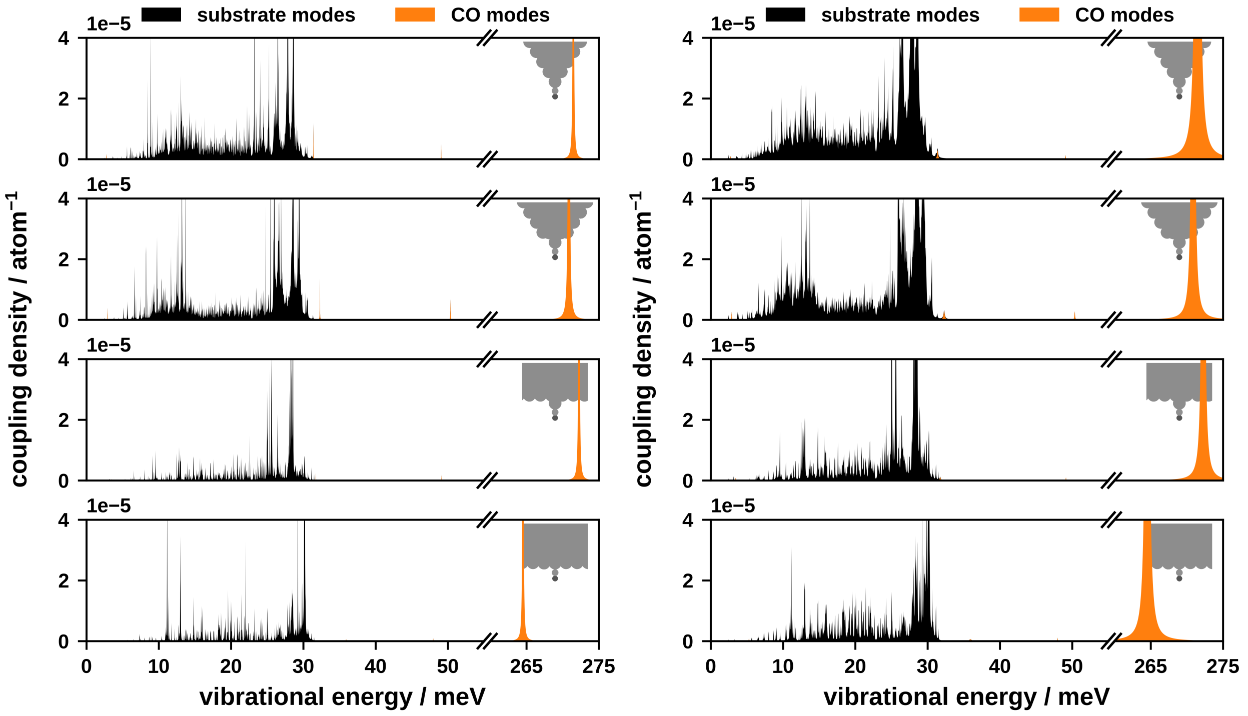

Figure S27: Coupling density of the IS mode with all other modes; Left) PPC only; Right) EPC and PPC.

References

(1) Blum, V.; Gehrke, R.; Hanke, F.; Havu, P.; Havu, V.; Ren, X.; Reuter, K.; Scheffler, M. Ab initio molecular simulations with numeric atom-centered orbitals. *Comput. Phys. Commun.* **2009**, *180*, 2175–2196.

(2) Ren, X.; Rinke, P.; Scheffler, M. Exploring the random phase approximation: Application to CO adsorbed on Cu(111). *Phys. Rev. B* **2009**, *80*, 045402.

(3) Feibelman, P. J.; Hammer, B.; Nørskov, J. K.; Wagner, F.; Scheffler, M.; Stumpf, R.; Watwe, R.; Dumesic, J. The co/pt (111) puzzle. *The Journal of Physical Chemistry B* **2001**, *105*, 4018–4025.

(4) Hu, Q.-M.; Reuter, K.; Scheffler, M. Towards an Exact Treatment of Exchange and Correlation in Materials: Application to the "CO Adsorption Puzzle" and Other Systems. *Phys. Rev. Lett.* **2007**, *98*, 176103.

(5) Kessler, J.; Thieme, F. Chemisorption of CO on differently prepared Cu(111) surfaces. *Surf. Sci.* **1977**, *67*, 405–415.

(6) Vollmer, S.; Witte, G.; Wöll, C. Determination of site specific adsorption energies of CO on copper. *Catalysis letters* **2001**, *77*, 97–101.

(7) Eren, B.; Zherebetskyy, D.; Patera, L. L.; Wu, C. H.; Bluhm, H.; Africh, C.; Wang, L.-W.; Somorjai, G. A.; Salmeron, M. Activation of Cu (111) surface by decomposition into nanoclusters driven by CO adsorption. *Science* **2016**, *351*, 475–478.

(8) Braun, J.; Graham, A. P.; Hofmann, F.; Silvestri, W.; Toennies, J. P.; Witte, G. A He-atom scattering study of the frustrated translational mode of CO chemisorbed on defects on copper surfaces. *J. Chem. Phys.* **1996**, *105*, 3258–3263.

(9) Hirschmugl, C. J.; Williams, G. P. Chemical shifts and coupling interactions for the bonding vibrational modes for CO/Cu (111) and (100) surfaces. *Phys. Rev. B* **1995**, *52*, 14177.

(10) Batatia, I.; Kovacs, D. P.; Simm, G.; Ortner, C.; Csányi, G. MACE: Higher order equivariant message passing neural networks for fast and accurate force fields. *Advances in Neural Information Processing Systems* **2022**, *35*, 11423–11436.

(11) Pettenkofer, C.; Otto, A. "Chemical effects" of vibrational lifetime and frequency of CO ON Ag. *Surface Science* **1985**, *151*, 37–51.

(12) Abe, H.; Manzel, K.; Schulze, W.; Moskovits, M.; DiLella, D. Surface-enhanced Raman spectroscopy of CO adsorbed on colloidal silver particles. *The Journal of Chemical Physics* **1981**, *74*, 792–797.

(13) Hrbek, J.; Hoffmann, F. M.; Park, J. B.; Liu, P.; Stacchiola, D.; Hoo, Y. S.; Ma, S.; Nambu, A.; Rodriguez, J. A.; White, M. G. Adsorbate-driven morphological changes of a gold surface at low temperatures. *Journal of the American Chemical Society* **2008**, *130*, 17272–17273.

(14) Li, W.-L.; Lininger, C. N.; Chen, K.; Vaissier Welborn, V.; Rossomme, E.; Bell, A. T.; Head-Gordon, M.; Head-Gordon, T. Critical role of thermal fluctuations for co binding on electrocatalytic metal surfaces. *JACS Au* **2021**, *1*, 1708–1718.

(15) Lončarić, I.; Alducin, M.; Juaristi, J. I.; Novko, D. CO Stretch Vibration Lives Long on Au(111). *The Journal of Physical Chemistry Letters* **2019**, *10*, 1043–1047.

(16) Shirhatti, P. R.; Rahinov, I.; Golibrzuch, K.; Werdecker, J.; Geweke, J.; Altschäffel, J.; Kumar, S.; Auerbach, D. J.; Bartels, C.; Wodtke, A. M. Observation of the adsorption and desorption of vibrationally excited molecules on a metal surface. *Nature chemistry* **2018**, *10*, 592–598.

(17) Forsblom, M.; Persson, M. Vibrational lifetimes of cyanide and carbon monoxide on noble and transition metal surfaces. *The Journal of chemical physics* **2007**, *127*.

(18) Pischel, J.; Pucci, A. Low-temperature adsorption of carbon monoxide on gold surfaces: IR spectroscopy uncovers different adsorption states on pristine and rough Au (111). *The Journal of Physical Chemistry C* **2015**, *119*, 18340–18351.
